# Inhibition of focal adhesion kinase impairs tumor formation and preserves hearing in a murine model of NF2-related schwannomatosis

**DOI:** 10.1126/sciadv.ady8382

**Published:** 2026-01-30

**Authors:** Dana K. Mitchell, Kylee Brewster, Li Jiang, Henry Mang, Waylan K. Bessler, Xiaohong Li, Qingbo Lu, Shaomin Qian, Eric York, Sarah K. Morrow, Shelley A. H. Dixon, Christopher Davis, Ka-Kui Chan, Abbi Smith, Alyssa C. Flint, Victor V. Le, Anna Geisinger, Francis Enane, Behnam Nabet, Steven D. Rhodes, Steven P. Angus, D. Wade Clapp

**Affiliations:** ^1^ Department of Pediatrics, Herman B Wells Center for Pediatric Research, Indiana University School of Medicine, Indianapolis, IN, USA.; ^2^ Indiana Biosciences Research Institute, Indianapolis, IN, USA.; ^3^ Human Biology Division, Fred Hutchinson Cancer Center, Seattle, WA, USA.; ^4^ Department of Pharmacology, University of Washington, Seattle, WA, USA.; ^5^ Department of Medical and Molecular Genetics, Indiana University School of Medicine, Indianapolis, IN, USA.; ^6^ Division of Pediatric Hematology/Oncology/Stem Cell Transplant, Indiana University School of Medicine, Indianapolis, IN, USA.; ^7^ IU Simon Comprehensive Cancer Center, Indiana University School of Medicine, Indianapolis, IN, USA.; ^8^ Department of Pharmacology and Toxicology, Indiana University School of Medicine, Indianapolis, IN, USA.; ^9^ Department of Biochemistry and Molecular Biology, Indiana University School of Medicine, Indianapolis, IN, USA.

## Abstract

NF2 (neurofibromatosis type 2)–related schwannomatosis (NF2-SWN) is a cancer predisposition syndrome characterized by the development of bilateral vestibular (VS) and spinal schwannomas. While benign, these tumors can cause substantial morbidity, and effective pharmacological treatments remain limited. Here, we demonstrate that genetic ablation of focal adhesion kinase (*Fak/Ptk2*) impairs tumor formation and preserves hearing in a murine model of NF2. Mechanistically, we show that *Fak* deletion decreases macrophage infiltration, attenuates nucleotide-binding oligomerization domain–containing protein 2–, leucine rich repeats (LRR)- and pyrin domain–containing protein 3 inflammasome activation, and suppresses the hepatocyte growth factor–MET axis. Pharmacological inhibition of FAK with single agent VS-4718 did not significantly reduce macroscopic tumor volume; however, its use in combination with the mitogen-activated protein kinase kinase (MEK) inhibitor selumetinib resulted in both a significant reduction in tumor volume and the preservation of dorsal root ganglion architecture. Our findings establish a critical role for FAK in schwannoma development and provide rationale for evaluation of combination FAK plus MEK inhibition in future clinical trials for NF2-associated SWN.

## Teaser

Inhibition of focal adhesion kinase (FAK) impairs schwannoma formation and preserves hearing in an NF2 mouse model.

## INTRODUCTION

NF2 (neurofibromatosis type 2)–related schwannomatosis (NF2-SWN; formerly called neurofibromatosis type 2) is an autosomal dominant cancer predisposition syndrome caused by germline haploinsufficiency of the *NF2* tumor suppressor gene (*1*). A hallmark of NF2-SWN is the development of bilateral vestibular (VS) and spinal schwannomas secondary to loss of heterozygosity of *NF2* in Schwann cells (SCs) and their precursors (*1*). Individuals without NF2-SWN can also develop sporadic vestibular schwannomas (sVS), and more than 90% of sVS exhibit biallelic disruption of *NF2* in the tumorigenic cells, suggesting a role for *NF2* loss in driving both genetic and sporadic disease (*2*). While benign, VS are associated with notable morbidity (*1*, *2*). Compromise of cranial and spinal nerve function due to compression or inflammation can cause substantial neurological deficiencies including hearing loss, vertigo, facial paralysis, chronic neuropathic pain, and even death (*1*, *2*). Surgical excision remains the standard of care, but due to nerve involvement and proximity to adjacent vital structures, this can be associated with major risks (*3*). At present, no long-term effective therapies have been identified, and the development of pharmacologic approaches that halt or reverse the growth of these tumors remains a critical unmet need.

Work by our group recently identified the multi–receptor tyrosine kinase (RTK) inhibitor brigatinib as an agent capable of reducing both schwannoma size and number in our murine model of NF2-SWN (*4*). While brigatinib is known to inhibit a number of kinases, using multiplexed kinase inhibitor beads and quantitative mass spectrometry (MIB/MS), we identified focal adhesion kinase (FAK; encoded by the *PTK2* gene) as a robustly inhibited kinase both in vitro and in vivo following brigatinib treatment (*4*). A phase 2 clinical trial based on this work was recently completed and demonstrated clinical benefit of brigatinib in heavily treated patients with NF2-SWN–associated tumors (*5*). Notably, brigatinib treatment was also associated with hearing improvement in 35% of eligible ears and a decrease in self-reported pain (*5*). Similarly, crizotinib, a US Food and Drug Administration (FDA)–approved inhibitor of anaplastic lymphoma kinase (ALK), has also been shown to inhibit FAK and has demonstrated preclinical efficacy against NF2-SWN–associated schwannomas (*6*, *7*). Accordingly, crizotinib is currently under evaluation for the treatment of NF2-SWN VS in an active clinical trial (NCT04283669). While brigatinib and crizotinib show promise for the treatment of NF2-SWN–associated schwannoma, both agents exhibit notable polypharmacology, which can exacerbate off-target effects and toxicity, potentially limiting their long-term use (*8*, *9*). Therefore, the identification of next-generation targeted therapeutics with increased specificity and improved tolerability is crucial for improving outcomes in this vulnerable patient population.

FAK has been shown to promote tumor formation and progression via both kinase-dependent and -independent functions in a range of solid tumors (*10*). In addition, emerging data suggest that FAK exerts it protumorigenic effects by regulating both tumor intrinsic and microenvironmental processes (*10*). While prior work suggests a role for FAK in NF2-SWN–associated tumors, studies have yet to evaluate whether targeted inhibition of FAK alone is necessary and sufficient to inhibit schwannoma initiation or progression in vivo (*6*, *7*, *11*). Furthermore, the mechanisms by which FAK contributes to schwannoma genesis remain elusive.

In the present study, we demonstrate that genetic ablation of *Fak (Ptk2)* impairs VS tumor formation and preserves hearing in a murine model of NF2-SWN. We show that *Fak* loss decreases macrophage infiltration, suppresses nucleotide-binding oligomerization domain–containing protein 2 (NOD2)–, leucine rich repeats (LRR)- and pyrin domain–containing protein 3 (NLRP3) inflammasome activation, and attenuates the hepatocyte growth factor (HGF)–MET signaling axis. While the use of a highly specific, clinical grade FAK inhibitor (VS-4718) did not decrease macroscopic tumor volume as a single agent, its use in combination with the mitogen-activated protein kinase (MAPK) kinase (MEK) inhibitor selumetinib resulted in a significant reduction in tumor volume and preservation of dorsal root ganglion (DRG) architecture in vivo. Collectively, our findings provide critical insight into the role of FAK in schwannoma genesis and support the evaluation of combinatorial strategies involving FAK inhibition in future clinical trials.

## RESULTS

### Genetic targeting of *Fak* impairs tumor formation and preserves hearing in a murine model of NF2-SWN

**Fig. 1.**
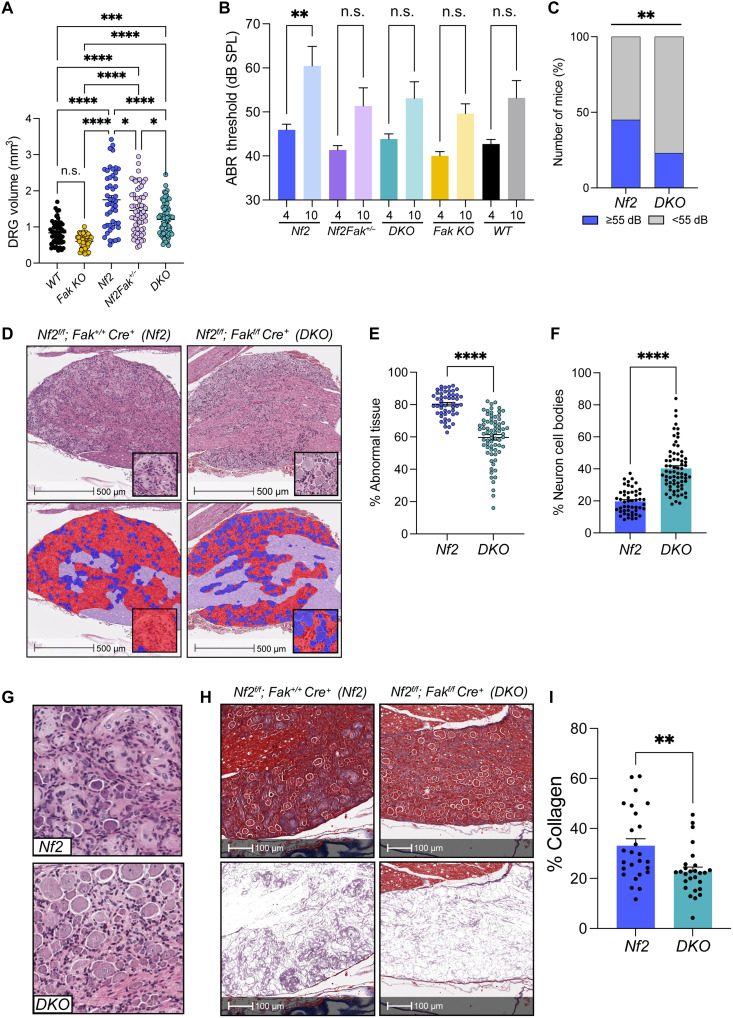
Genetic targeting of *Fak* impairs tumor formation and preserves hearing in a murine model of NF2-SWN. (**A**) Scatter plot depicting DRG volumes (in cubic millimeters) from 10-month-old mice of the indicated genotypes (*WT*, *n* = 14; *Fak KO*, *n* = 12; *Nf2*, *n* = 12; *Nf2Fak^+/−^*, *n* = 16; *DKO n* = 18). Dots represent individual DRG (*n* = 4 per mouse). Error bars reflect the SEM. *P* values represent one-way analysis of variance (ANOVA) with Tukey’s multiple-comparison test. (**B**) Bar plot depicting the average ABR threshold measured in decibels of mice with the indicated genotypes at 4 and 10 months of age (*WT*, *n* = 11; *Fak KO*, *n* = 13; *Nf2*, *n* = 11; *Nf2Fak^+/−^*, *n* = 11; *DKO n* = 13). *P* values represent one-way ANOVA with Šídák’s multiple-comparison test. Error bars reflect the SEM. (**C**) Bar plot depicting a ~2-fold RR of hearing impairment (average ABR threshold ≥ 55 dB) at 10 months of age among *Nf2* and *DKO* mice. RR = 1.957, 95% CI (1.301 to 2.992), and *P* = 0.0016. *P* value reflects two-sided, Fisher’s exact test. (**D**) Representative photomicrographs of hematoxylin and eosin (H&E)–stained DRG obtained from 10-month-old *Nf2* and *DKO* mice. Mask in the bottom panel and depicts abnormal tissue (red), neuron cell bodies (blue), and nerve (lavender). (**E** and **F**) Plots comparing the percentage of abnormal tissue (E) and neuron cell bodies (F) in *Nf2* and *DKO* DRGs. Dots represent individual DRG. Error bars reflect the SEM. *P* values represent unpaired, two-tailed *t* tests between groups. (**G**) Representative photomicrographs of H&E-stained tissue sections comparing the architecture of *Nf2* and *DKO* DRGs. (**H**) Representative photomicrographs of Masson’s trichrome–stained DRG obtained from *Nf2* and *DKO* mice. Mask in the bottom panel and depicts collagen staining in purple. (**I**) Plot comparing collagen within the 10-month-old *Nf2* and *DKO* DRGs, reflected as percentage of the total tissue area analyzed. Dots represent individual DRG. Error bars reflect the SEM. *P* values represent unpaired, two-tailed *t* test. *P ≤ 0.05, **P ≤ 0.01, ***P ≤ 0.001, and ****P ≤ 0.0001.

*Nf2^flox/flox^;Postn-Cre^+^(Nf2)* mice harbor loss of *Nf2* in SCs and their precursors. These mice develop spontaneous, cranial nerve V (CN V), CN VIII, and paraspinal schwannomas within the DRG that are histologically consistent with human disease (*12*, *13*). In addition, *Nf2* mice also recapitulate the progressive hearing loss exhibited by human patients with VS. Therefore, to interrogate whether genetic disruption of *Fak* (*Ptk2*, hereafter referred to as *Fak*) can prevent schwannoma genesis or VS-associated progressive hearing loss, we generated *Nf2^flox/flox^; Fak ^flox/flox^;Postn-Cre^+^* [*DKO* (double knockout)] mice, characterized by the conditional loss of both *Nf2* and *Fak* within schwannoma cells of origin. As *Nf2* mice develop tumors, DRG volume increases and serves as a surrogate for tumor volume. Macroscopic comparison of DRG from *Nf2* and *DKO* mice revealed that genetic ablation of *Fak* significantly decreased DRG volume in a gene dose–dependent manner (Fig. 1A). Evaluation of auditory brainstem reflex (ABR) thresholds at 4 and 10 months of age demonstrated that both hetero- and homozygous deletion of *Fak* protected against hearing loss to a degree consistent with that seen in wild-type (WT) mice (Fig. 1B). Compared to *DKO* mice, *Nf2* mice were nearly twice [relative risk (RR) = 1.957, 95% confidence interval (CI; 1.301 to 2.992), and *P* = 0.0016] as likely to be hearing impaired (ABR threshold > 55 dB) at 10 months of age (Fig. 1C).

The DRGs of WT mice are histologically characterized by tightly packed clusters of large, round neuronal cell bodies surrounded by satellite glial cells (fig. S1A, left). By 8 months of age, 100% of *Nf2* mice will have developed tumors consistent with frank schwannoma and characterized predominantly by Antoni A areas and onion-bulb formations (OBFs) (fig. S1A, right) (*12*, *13*). Accordingly, analysis of hematoxylin and eosin (H&E)–stained slides using the Indica Labs HALO DenseNet AI module (Fig. 1D) revealed a significant decrease in the percentage of abnormal tissue (Fig. 1E) and a significant increase in the percentage of neuron cell bodies (Fig. 1F) within the DRG of *DKO* mice. This suppression of tumor growth and maintenance of normal tissue architecture was sustained in *DKO* DRG until at least 16 months of age (fig. S1, B to E). The abnormal tissue within the *Nf2* DRG consisted predominantly of Antoni A areas and onion-bulb formations, whereas that within *DKO* DRG was primarily characterized by hypercellularity (Fig. 1G). OBFs, which describe concentric layers of SC processes and collagen deposition surrounding axons, occur secondary to repeated cycles of demyelination and remyelination. Therefore, the presence of OBFs reflects a chronic, unresolved injury-like phenotype (*14*, *15*). To quantify the extent of OBFs in *Nf2* and *DKO* DRG, we performed quantitative analysis of trichrome staining, which demonstrated a significant decrease in collagen deposition within both the 10- and 16-month-old *DKO* DRG, suggesting that OBFs were less prevalent in DRG obtained from *DKO* mice (Fig. 1, H and I, and fig. S1, F and G).

Loss of *NF2* has been shown to promote SC proliferation and dedifferentiation, whereas *FAK* deficiency in SCs has the opposite effect (*16*–*18*). While both *Nf2*- and *Fak*-null SCs have been shown to exhibit defective myelination, the latter is thought to occur secondary to insufficient SC numbers (*19*). *DKO* mice did not exhibit any overt developmental abnormalities or evidence of gross myelination defects. Quantification of nuclei within the peripheral nerves revealed that, while the peripheral nerves of *DKO* mice were characterized by significantly fewer nuclei than those from *Nf2* mice, this did not achieve levels below those of *WT* mice (fig. S2, A and B). Similarly, quantification of SRY-box transcription factor 10 (SOX10), a marker of SCs within the DRG, also revealed a significant decrease in the number of SOX10^+^ cells within *DKO* compared to *Nf2*, but not *WT* DRG (fig. S2, C and D) (*20*). Collectively, these findings suggest that in the setting of NF2 deficiency, the conditional ablation of *Fak* in SCs and their precursors is sufficient to impair schwannoma growth, maintain normal DRG architecture, and preserve hearing without compromising myelination or normal development.

### Genetic inhibition of *Fak* attenuates the inflammatory signaling in vivo

**Fig. 2.**
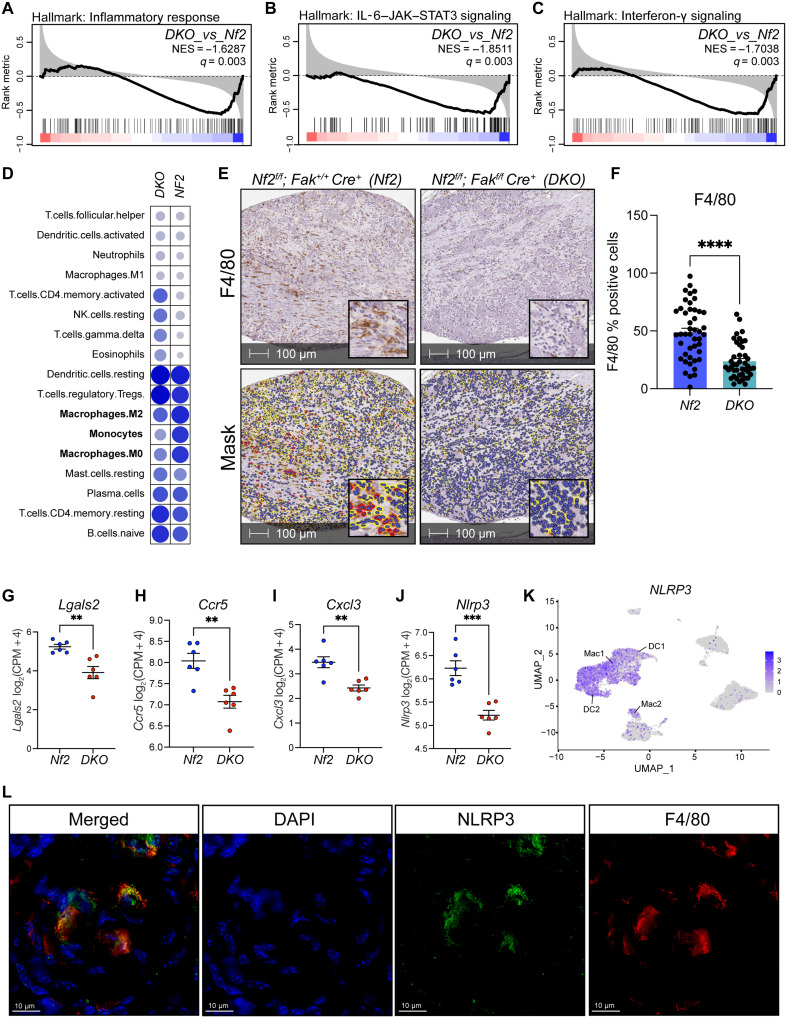
Genetic inhibition of *Fak* attenuates the inflammatory signaling in vivo. (**A** to **C**) Gene set enrichment plots depicting negative enrichment of the Hallmark inflammatory response (A), IL-6–JAK–STAT3 (B), and interferon-γ signaling (C) gene signatures by *DKO* DRGs. Black vertical bars indicate the rank of genes comprising the gene signature. Black curve corresponds to the running enrichment score for the gene set. Normalized enrichment score (NES) and *q* value for *DKO* compared to *Nf2* DRG are as shown. (**D**) Cell type mapping panel representing inferred cell types in *Nf2* and *DKO* DRG. (**E**) Representative photomicrographs of DRG immunohistochemically stained for F4/80. HALO cytonuclear mask is shown in the bottom panel. Blue cells indicate negative staining for F4/80, yellow cells indicate weak positive (1+), orange cells indicate moderate positive (2+), and red cells indicate strong positive (3+). (**F**) Bar plot depicting the percentage (%) of cells exhibiting positive staining of F4/80. Dots indicate individual DRG. Error bars reflect the SEM. *P* value represents unpaired, two-tailed *t* test. (**G** to **J**) Scatter plots comparing log_2_[counts per million (CPM) + 4] normalized counts of *Lgals2* (G), *Ccr5* (H), *Cxcl3* (I), and *Nlrp3* (J) in *Nf2* and *DKO* DRG. Dots represent individual mice. Error bars reflect the SEM. *P* values represent unpaired, two-tailed *t* test. (**K**) Uniform manifold approximation and projection (UMAP) visualization depicting the expression of *NLRP3* across distinct cell populations in human VS. Color overlays indicate *NLRP3* transcript levels per cell. Gradient of gray to indigo indicates relative abundance of transcripts per cell, with indigo indicating higher expression and gray signifying a lack of detection. (**L**) Representative images of IF staining of F4/80 (red) and NLRP3 (green) in a murine DRG. 4′,6-diamidino-2-phenylindole (DAPI) is blue. *P ≤ 0.05, **P ≤ 0.01, ***P ≤ 0.001, and ****P ≤ 0.0001. Macrophages (Mac1 and Mac2) and dendritic cells (DC1 and DC2).

To better understand the mechanisms underlying the phenotypic differences observed above, we performed bulk RNA sequencing (RNA-seq) on DRGs obtained from *Nf2* and *DKO* mice. Hallmark gene set enrichment analysis (GSEA) revealed suppression of inflammatory pathways in DRG obtained from *DKO* mice (Fig. 2A). Specifically, *DKO* samples exhibited decreased interleukin-6 (IL-6)–Janus kinase (JAK)–signal transducer and activator of transcription 3 (STAT3) (Fig. 2B) and interferon-γ signaling (Fig. 2C). Cell type profiling using computational deconvolution methods and LM22 (*21*) as the reference dataset demonstrated decreased enrichment of gene signatures consistent with macrophage and monocyte infiltration in *DKO* compared to *Nf2* DRG (Fig. 2D). Confirmatory immunohistochemical staining showed a significant decrease in the staining of murine macrophage marker F4/80 in *DKO* DRG (Fig. 2, E and F). Accordingly, differential gene expression (DEG) analysis revealed down-regulation of genes known to be involved in the regulation of macrophage and monocyte immune responses including *Lgals2*, *Ccr5*, *Cxcl3*, and *Nlrp3* (Fig. 2, G to J) (*22*–*25*). Complementary single-cell RNA-seq (scRNA-seq) analysis of publicly available human NF2-associated VS revealed expression of *Cxcl3 and Nlrp3* to be largely restricted to macrophages, whereas *Ccr5* was expressed by both macrophages and T cells (Fig. 2K and fig. S3, A to D). Immunofluorescent (IF) imaging of *Nf2* DRGs confirmed colocalization between murine macrophage marker F4/80 and NLRP3 (Fig. 2L).

**Fig. 3.**
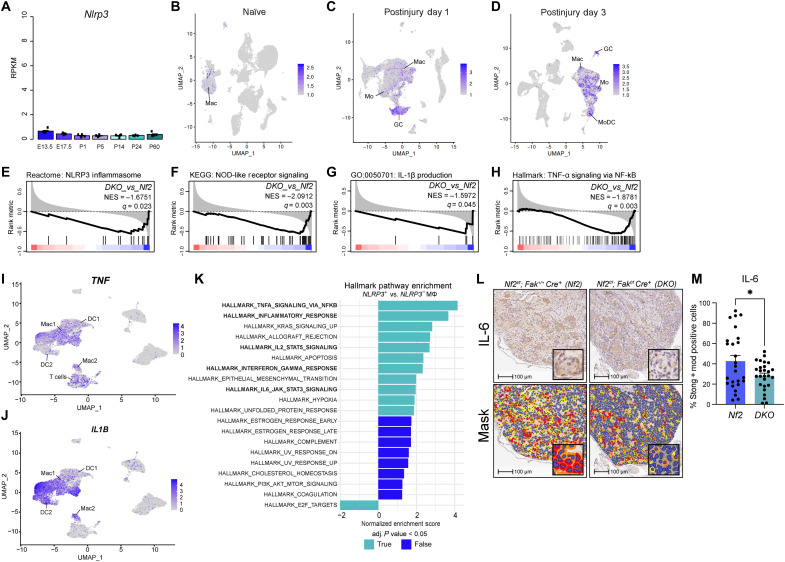
Genetic inhibition of *Fak* suppresses the NLRP3 inflammasome. (**A**) Bar plot of *Nlrp3* transcript expression [reads per kilobase of transcript per million mapped reads (RPKM)] at indicated days of embryonic [embryonic day 13.5 (E13.5) and E17.5] and postnatal [postnatal day 1 (P1), P5, P14, P24, and P60] development. (**B** to **D**) UMAPs depicting the expression of *Nlrp3* across distinct cell populations in the murine sciatic nerve 1 (C) and 3 (D) days postinjury. *Nlrp3* expression in the naïve murine sciatic nerve serves as a reference (B). (**E** to **H**) Gene set enrichment plots depicting negative enrichment of the indicated gene signatures by *DKO* DRG. Black vertical bars indicate the rank of genes comprising the gene signature. Black curve corresponds to the running enrichment score for the gene set. NES and *q* value reflect *DKO* compared to *Nf2* DRG. GO, Gene Ontology. (**I** and **J**) UMAPs depicting the expression of *TNF* (I) and *IL-1B* (J) across distinct cell populations in human VS. (**K**) Bar plot depicting Hallmark pathway enrichment analysis of the top 20 biological pathways enriched in *NLRP3^+^* compared to *NLRP3*^–^ macrophages in human VS. X-axis represents the NES, with positive values indicating up-regulation and negative values indicating down-regulation. Pathways are ranked by significance. Turquoise bars represent pathways that meet the significance threshold (adjusted *P* < 0.05). adj., adjusted. (**L**) Representative photomicrographs of DRG immunohistochemically stained for IL-6. The HALO cytonuclear mask is shown in the bottom panel. Blue cells indicate negative staining for IL-6, yellow cells indicate weak positive (1+), orange cells indicate moderate positive (2+), and red cells indicate strong positive (3+). (**M**) Bar plot depicting the percentage of cells exhibiting strong and moderate staining of IL-6. Dots indicate individual DRG. Error bars reflect the SEM. *P* value represents unpaired, two-tailed *t* test. Color overlays indicate transcript levels per cell. Gradient of gray to indigo indicates relative abundance of transcripts per cell, with indigo indicating higher expression and gray signifying a lack of detection. mod, moderate. *P ≤ 0.05.

*Nlrp3* encodes the NLRP3, which serves as the sensor for the NLRP3 inflammasome and is responsible for the recognition of endogenous danger signals, microbes, and other noxious environmental stimuli (*26*, *27*). Data from the Sciatic Nerve Atlas (SNAT) demonstrate that during homeostatic conditions, the expression of *Nlrp3* is quite low in the murine peripheral nervous system (Fig. 3A) (*28*). Conversely, in the days immediately following sciatic nerve injury (postinjury days 1 and 3), the expression of *Nlrp3* is increased, further suggesting the persistence of a nerve injury–like state in *Nf2* DRG (Fig. 3, B to D, and fig. S4, A to C). Upon activation, the NLRP3 inflammasome forms in macrophages and consistent with their decreased infiltration in *DKO* DRG detailed above, gene signature enrichment analysis found *DKO* DRG to be characterized by decreased NLRP3 inflammasome activity (Fig. 3E). Activation of the NLRP3 inflammasome can occur via several mechanisms including the recognition of pathogen- or damage-associated molecular patterns by NOD2 or through IL-1β and tumor necrosis factor–α (TNFα)–mediated stimulation of nuclear factor κB (NF-κB) (*26*). Accordingly, *DKO* DRG exhibited negative enrichment of signatures consistent with NOD2 receptor (Fig. 3F), IL-1β (Fig. 3G), and TNF signaling (Fig. 3H). Analysis of scRNA-seq data from human NF2-SWN–associated VS identified macrophages as the predominant source of *TNF* (Fig. 3I) and *IL1B* (Fig. 3J). As expected, GSEA comparing NLRP3-positive (NLRP3^+^) to NLRP3-negative (NLRP3^–^) macrophages revealed a significant, positive enrichment of the Hallmark TNF-α signaling via NF-κB gene signature by NLRP3^+^ macrophages (Fig. 3K). Activation of NF-κB and other IL-1β and TNFα-mediated pathways promotes an increase in the expression of inflammatory cytokines including IL-6 (*29*). Consistent with our results indicating suppression of these pathways, immunohistochemical analysis revealed a significant decrease of IL-6 in *DKO* DRG, suggesting that *Fak* inhibition suppresses inflammatory signaling within the VS microenvironment (Fig. 3, L and M).

### *Fak* inhibition suppresses HGF-MET signaling in vivo

**Fig. 4.**
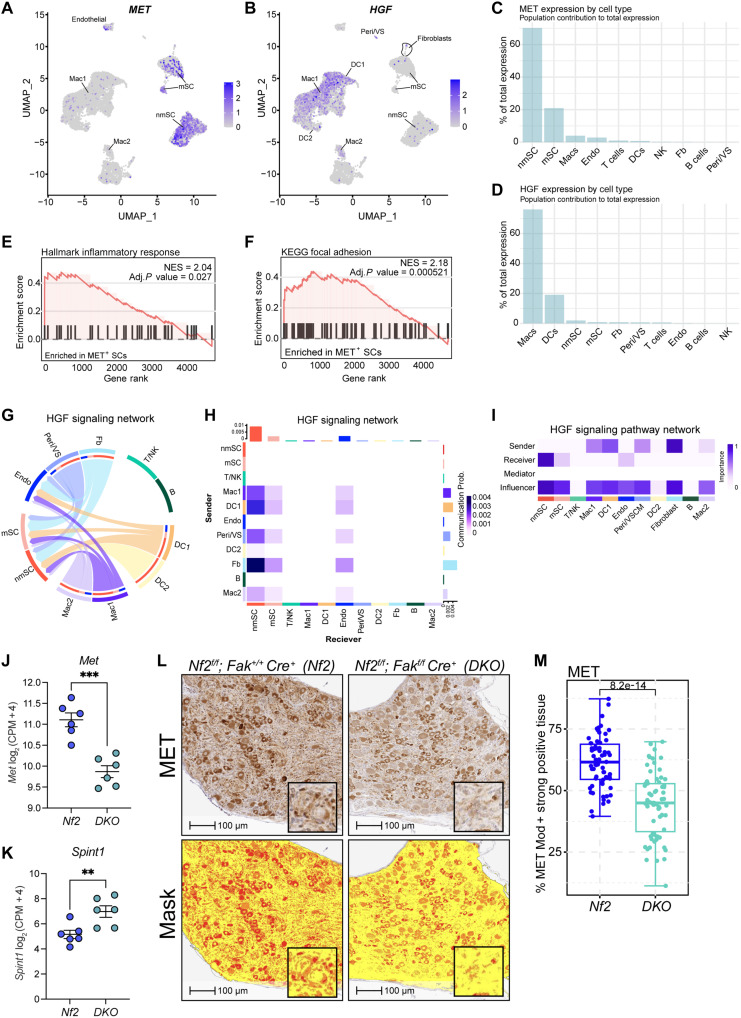
*Fak* inhibition suppresses HGF-MET signaling in vivo. (**A** and **B**) UMAPs depicting the expression of *MET* (A) and *HGF* (B) in human VS. Color overlays indicate *MET* or *HGF* transcript levels per cell. Gradient of gray to indigo indicates relative abundance of transcripts per cell, with indigo indicating higher expression and gray signifying a lack of detection. (**C** and **D**) Bar plot depicting the weighted contributions of *MET* (C) and *HGF* (D) expression across distinct cell populations in human VS. (**E** and **F**) GSEA plots depicting positive enrichment of the indicated pathways by *MET*^+^ SCs in human VS. NES and adjusted *P* values are shown. (**G**) Circle plot depicting the HGF signaling network. Connecting ribbons reflect predicted signaling interactions, with ribbon width proportional to interaction strength and arrows indicating directionality. (**H**) Heatmap depicting the communication probability scores between cell pairs within the HGF signaling network. Gradient indicates the strength of predicted cell-cell communication. Prob., probability. (**I**) Matrix depicting cellular functional classification in the HGF signaling network. Color intensity reflects the importance score for each role, with 1 signifying the highest importance and 0 the lowest. (**J** and **K**) Scatter plots comparing expression of *Met* (J) and *Spint1* (K) in *Nf2* and *DKO* DRG. Dots represent individual mice. Error bars reflect the SEM. *P* values represent unpaired, two-tailed *t* test. (**L**) Photomicrographs of DRG stained for MET. HALO mask is shown in the bottom panel. Yellow indicates weak positive (1+), orange indicates moderate positive (2+), and red indicates strong positive (3+) staining for MET. (**M**) Box and whisker plot depicting MET protein expression in DRG obtained from *Nf2* and *DKO* mice. Dots represent individual DRG. Error bars represent the 95% CI. *P* value represents unpaired, two-tailed *t* test. Nonmyelinating SC (nmSC), myelinating SC (mSC), fibroblasts (Fb), macrophages (Mac1 and Mac2), endothelial (Endo), natural killer/T (NK/T), dendritic (DC1, DC2), B (B), and perivascular/vascular smooth muscle (Peri/VS) cells. **P ≤ 0.01 and ***P ≤ 0.001.

Prior work by our group demonstrated that compared to normal nerve, the expression of MET and its active form, phospho-MET Tyr^1234/1235^ (Y1234/Y1235), is significantly increased in human VS (*30*). Consistent with this finding, functional kinome profiling of human SCs revealed MET and Ephrin type-B receptor 2 (EPHB2) to be the most up-regulated kinases in NF2-deficient SCs compared to the WT control (fig. S5). Notably, FAK is a downstream effector of the ephrin receptors including EPHB2, which is known to play a critical role in the migration and organization of SCs during nerve regeneration and repair (*31*, *32*). In line with this and our above findings, recent work by Barrett *et al.* identified a subset of SCs within VS (VS-SCs) that are characterized by an injury-like phenotype, exhibit elevated expression of *MET*, and are associated with an increased infiltration of myeloid lineage cells (*33*, *34*). Accordingly, the HGF-MET axis has been shown to become particularly active in SCs in response to nerve injury, during which it promotes SC dedifferentiation and proliferation (*35*). Analysis of data from the Injured SNAT (iSNAT) revealed that activation of the HGF-MET signaling axis occurs in the days following injury of the murine sciatic nerve and that *Met*-expressing SCs serve as the recipients of HGF supplied predominantly by infiltrating macrophages (fig. S6, A to F) (*36*). Analysis of scRNA-seq data from human NF2-SWN–related VS revealed similar results, identifying SCs as the major source of *MET* expression and macrophages as the primary supplier of the MET receptor ligand *HGF* (Fig. 4, A to D). GSEA comparing MET-positive (MET^+^) to MET-negative (MET^–^) SCs revealed significant, positive enrichment of the Hallmark inflammatory response (Fig. 4E) and the Kyoto Encyclopedia of Genes and Genomes (KEGG) Focal Adhesion (Fig. 4F) gene signatures by MET+ SCs, suggesting a link between MET, FAK, and the inflammatory schwannoma tumor microenvironment (TME).

In agreement with the murine data obtained from iSNAT, CellChat analysis of the human VS scRNA-seq data also identified SCs as the primary recipients of HGF supplied by cells, including macrophages, within the human VS TME (Fig. 4, G to I). Given that HGF stimulation can up-regulate the expression of MET, we hypothesized that the decreased macrophage infiltration noted in the *DKO* DRG would be accompanied by a decrease in the expression of *Met* (*37*). Consistent with our hypothesis, analysis of bulk RNA-seq from *Nf2* and *DKO* DRG revealed a significant decrease in *Met* expression in DRG obtained from *DKO* mice (Fig. 4J). The expression of *Spint1/Hai-1*, a member of the Kunitz-type serine protease inhibitors that prevents the cleavage of pro-HGF to its active form through the inhibition of HGF activator (HGFAC), was significantly up-regulated in *DKO* DRG (Fig. 4K) (*38*). Confirmatory immunohistochemical staining validated that MET was decreased at the protein level in *DKO* compared to *Nf2* DRG (Fig. 4, L and M). A modest, yet significant, decrease in *Met* expression was also observed in vitro following 24 hours of treatment with FAK-targeting PROTACs, but not small molecule kinase inhibitors (fig. S7A). FAK-targeting PROTACs function by linking a FAK inhibitor to an E3 ligase, creating a heterobifunctional molecule that facilitates the ubiquitination and subsequent proteasomal degradation of the FAK protein, thereby achieving a pharmacological knockout effect similar to genetic deletion (*39*, *40*). Consistent with the findings from our genetic model, these results similarly indicate that direct and kinase-independent functions of FAK may be responsible for the observed decrease in *Met* expression following FAK inhibition. Together, these data suggest that the conditional ablation of *Fak* suppresses the HGF-MET signaling axis in VS through multiple, independent mechanisms including down-regulation of *Met*, attenuation of HGFAC-mediated activation of pro-HGF via *Spint1* up-regulation, and impaired infiltration of HGF-producing macrophages.

### Combined pharmacologic inhibition of FAK and MEK decreases tumor volume and preserves normal tissue architecture in vivo

**Fig. 5.**
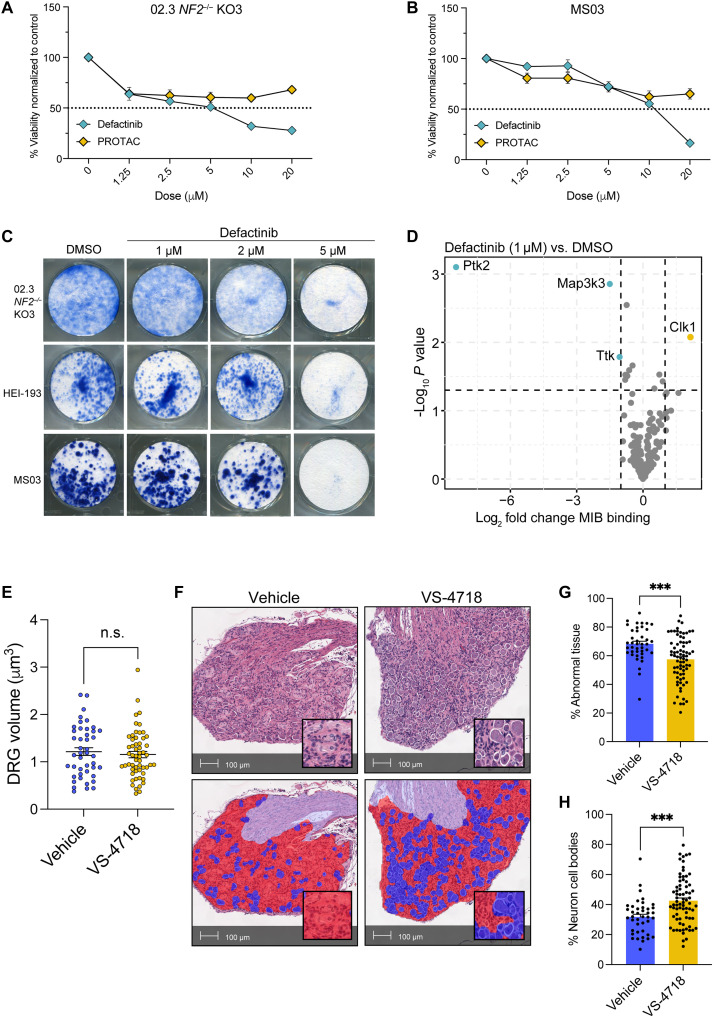
Pharmacologic inhibition of FAK in vitro. (**A** and **B**) Dose-response curves comparing the effects of defactinib and a FAK-targeting PROTAC on the viability in human (A) and murine (B) NF2-deficient SCs. Cells were treated with increasing concentrations (0 to 20 μM) of either defactinib or PROTAC for 48 hours, at which time viability was determined via CellTiter-Glo assay, and luminescent values were normalized to untreated control (100%). The dotted line indicates 50% viability. Data points represent mean values ± SEM of *n* = 12 (02.3 *NF2*^−/−^ KO3) or *n* = 4 (MSO3) replicates. (**C**) Colony formation assays stained with methylene blue and showing the effect of increasing concentrations of defactinib (1 to 5 μM) compared to dimethyl sulfoxide (DMSO) control on three NF2-deficient SC lines: 02.3 *NF2*^−/−^ K03, HEI-193, and MS03. (**D**) Volcano plot showing changes in MIB binding of kinases following 24-hour treatment with 1 μM defactinib compared to DMSO control in MS03 cells. *X* axis represents log_2_ fold change (FC) in binding, and the *y* axis shows the negative log_10_ of the *P* value. Dotted lines indicate significance thresholds for FC and *P* value. (**E**) Scatter plot depicting DRG volumes (in cubic millimeters) from mice treated with VS-4718 (*n* = 14) or vehicle control (*n* = 11). Dots represent individual DRG (*n* = 4 per mouse). *P* value represents unpaired, two-tailed *t* test. Error bars reflect the SEM. (**F**) Representative photomicrographs of H&E-stained DRG obtained from *Nf2* and *DKO* mice. HALO AI DenseNet mask is shown in the bottom panel and depicts abnormal tissue (red), neuron cell bodies (blue), and nerve (lavender). (**G** and **H**) Plots comparing the percentage of abnormal tissue (G) and neuron cell bodies (H) in *Nf2* and *DKO* DRG. Dots represent individual DRG. Error bars reflect the SEM. *P* values represent unpaired, two-tailed *t* test. ***P ≤ 0.001 and n.s., not significant.

On the basis of the promising results of our genetic intercross, we next pursued the evaluation of pharmacologic FAK inhibitors. Treatment of human (Fig. 5A) and murine (Fig. 5B) NF2-deficient SCs with the FAK-specific kinase inhibitor defactinib and a FAK-targeting PROTAC modestly impaired cell viability at doses above 1 μM in both lines. Colony-forming assay using human (02.3 *NF2*^−/−^ KO3 and HEI193) and murine (MS03) NF2-deficient SC lines revealed significant growth suppression in response to 5 μM defactinib (Fig. 5C). Notably, kinome profiling using MIB/MS found defactinib to be highly specific for FAK/protein tyrosine kinase 2 (PTK2) (Fig. 5D). Given the impact of the genetic ablation of *Fak* on the schwannoma microenvironment, we hypothesized that pharmacologic inhibition of FAK would prove more efficacious in vivo. Therefore, we treated *Nf2^flox/flox^;Postn-Cre^+^* (*Nf2*) mice with the FAK inhibitor VS-4718, which is used as a surrogate for defactinib in murine preclinical models due to its superior pharmacokinetic profile in mice (*41*). Treatment of MS03 cells with VS-4718 for 24 hours confirmed similar FAK specificity for VS-4718 and defactinib (fig. S8A). In contrast to the genetic inhibition of *Fak*, treatment with VS-4718 did not significantly reduce DRG volume (Fig. 5E). However, analysis of H&E-stained slides using the Indica Labs HALO DenseNet AI module (Fig. 5F) revealed a significant decrease in the percentage of abnormal tissue (Fig. 5G) and a significant increase in the percentage of neuron cell bodies (Fig. 5H) within the DRG of VS-4718–treated mice, suggesting that pharmacologic FAK inhibition resulted in a normalization of DRG architecture. In contrast to the results from our genetic model, we did not observe a significant decrease in macrophage infiltration or suppression of IL-6 in DRG obtained from VS-4718–treated mice (fig. S9, A to F). We did observe a significant increase in the protein levels of MET in VS-4718–treated DRGs, likely reflecting a compensatory up-regulation of MET following targeted FAK inhibition as the tumorigenic effects of MET have been shown to be mediated by FAK (fig. S9, G and H) (*42*). While these results contrast with those from the genetic model, they are in line with our RNA-seq results, indicating differences in the modulation of *MET* expression by kinase-dependent and -independent roles of FAK.

**Fig. 6.**
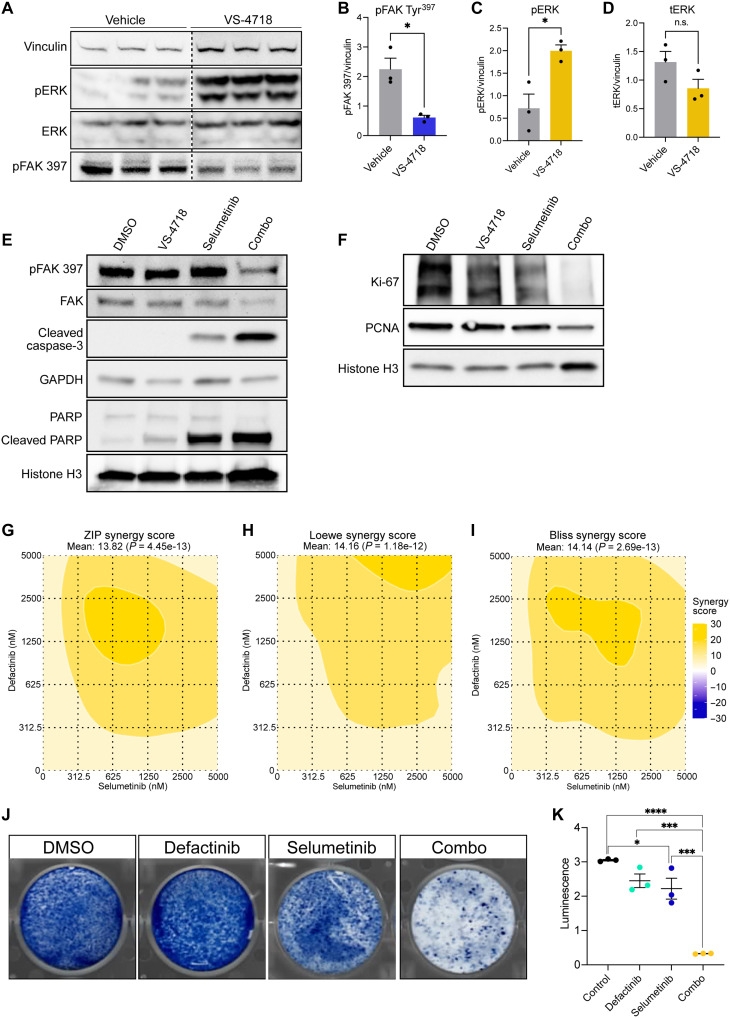
Combined pharmacologic inhibition of FAK and MEK is synergistic in vitro. (**A**) Western blot depicting pERK, total ERK, and phospho-FAK (pFAK) Tyr^397^ expression in nerves obtained from mice treated with VS-4718 (*n* = 3) or vehicle control (*n* = 3) for 2 weeks. Vinculin serves as a loading control. (**B** to **D**) Bar graphs quantifying pFAK Tyr^397^ (B), pERK (C), and total ERK (D) expression normalized to vinculin from the blots depicted in (A). Dots represent individual mice. Error bars reflect SEM. *P* value represents unpaired, two-tailed *t* test. tERK, total ERK. (**E** and **F**) Western blots depicting the expression of pFAK Tyr^397^, total FAK, cleaved caspase-3, and cleaved PARP (E), as well as the proliferation markers Ki-67 and PCNA (F) in a murine *Nf2*-deficient SC line (MS03) treated with 1 μM VS-4718 and selumetinib alone or in combination. DMSO serves as the control. Glyceraldehyde-3-phosphate dehydrogenase (GAPDH) and histone H3 are loading controls. (**G** to **I**) Heatmaps depicting Zero Interaction Potency (ZIP) (G), Loewe (H), and Bliss (I) synergy scores for combination defactinib plus selumetinib in murine NF2-deficient SCs (MS03). Viability (%) was evaluated at 48 hours by CellTiter-Glo and normalized to the untreated control. Data represent the mean of three replicates for each condition. Color scale indicates synergy scores from −30 (blue; antagonistic) to +30 (yellow; synergistic). Heatmaps and corresponding mean synergy scores were calculated with their respective statistical significance (ZIP: 13.82, *P* = 4.45 × 10^–13^; Loewe: 14.16, *P* = 1.18 × 10^–12^; Bliss: 14.14, *P* = 2.69 × 10^–13^) were computed using SynergyFinder+. (**J** and **K**) Colony formation assay stained with methylene blue (J) and dot plot (K) showing the effect of combination defactinib (1 μM) plus selumetinib (1 μM) compared to the single agents and DMSO control in a murine *Nf2*-deficient SC line (MS03). Dots represent independent samples. Error bars represent the SEM. *P* value represents unpaired, two-tailed *t* tests between groups. *P ≤ 0.05, ***P ≤ 0.001, ****P ≤ 0.0001, and n.s., not significant.

Western blot analysis of sciatic nerves obtained from VS-4718–treated mice also revealed significant up-regulation of phospho–extracellular signal–regulated kinase (ERK) (pERK) compared to vehicle controls (Fig. 6, A to D), indicating that compensatory activation of the MAPK signaling pathway may account for the diminished efficacy of VS-4718 in vivo. This is consistent with previous studies suggesting that long-term FAK inhibition drives hyperactivation of Ras/MAPK signaling (*43*). Similarly, MAPK pathway blockade has been shown to drive the reciprocal activation of FAK (*44*). These findings, in combination with prior work demonstrating synergy of combined FAK and RAF/MEK inhibition in other cancers, suggest that the combination of VS-4718 plus the MEK inhibitor selumetinib may exhibit increased efficacy in the treatment of NF2-SWN (*41*, *43*, *45*). In line with this hypothesis, Western blot analysis using a murine NF2-deficient schwannoma cell line (MS03) demonstrated that combination treatment with VS-4718 plus selumetinib significantly increased the levels of cleaved caspase-3 compared to either agent alone (Fig. 6E). Levels of cleaved poly(adenosine 5′-diphosphate–ribose) polymerase (PARP) were increased in all treatment groups compared to the dimethyl sulfoxide (DMSO) control, with a more robust increased noted in the selumetinib single agent and combination groups (Fig. 6E). Accordingly, we observed the most pronounced decrease in the levels of proliferation markers Kiel-67 antigen (Ki-67) and proliferating cell nuclear antigen (PCNA) in the combination group (Fig. 6F), suggesting that the combination of VS-4718 plus selumetinib exhibits both cytotoxic and cytostatic effects. Treatment of MS03 cells with defactinib plus selumetinib (Fig. 6, G to I) or the RAF/MEK inhibitor VS-6766 confirmed the synergistic activity of combined FAK and MEK inhibition in NF2-deficient cells (fig. S10, A to C). Consistent with these results, colony formation assay also revealed that the combination of defactinib plus selumetinib (Fig. 6, J and K) or VS-6766 (fig. S10D) was significantly more efficacious in suppressing cell proliferation than DMSO or any of the agents used alone.

**Fig. 7.**
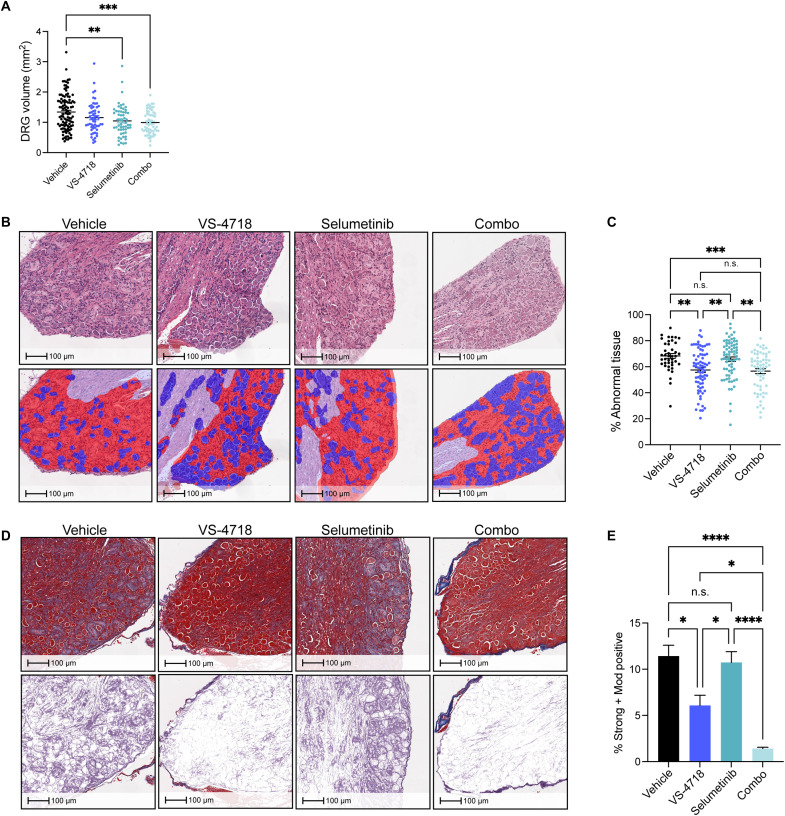
Combined pharmacologic inhibition of FAK and MEK decreases tumor volume and preserves normal tissue architecture in vivo. (**A**) Scatter plot depicting DRG volumes (in cubic millimeters) from mice treated with VS-4718 (*n* = 14) and selumetinib (*n* = 14), alone or in combination (*n* = 15) and compared to two cohorts (V1 and V2) of age- and sex-matched vehicle controls (V1: *n* = 11; V2: *n* = 15). Dots represent individual DRG (*n* = 4 per mouse). *P* values represent one-way ANOVA with Tukey’s multiple-comparison test. Error bars reflect SEM. DRG volumes (in cubic millimeters) for vehicles (V1: *n* = 11) and VS-4718 (*n* = 14) were initially presented in Fig. 5E and were included in this graph for the purpose of comparison. (**B**) Representative photomicrographs of H&E-stained DRG obtained from mice of the indicated treatment groups. Mask generated using Indica Lab’s HALO AI DenseNet module is shown in the bottom panel and depicts abnormal tissue (red), neuron cell bodies (blue), and nerve (lavender). (**C**) Scatter plot comparing the percentage of abnormal tissue in DRG of mice treated with VS-4718 (*n* = 12) and selumetinib (*n* = 14), alone or in combination (*n* = 12) and compared to age- and sex-matched vehicle controls (*n* = 7). Dots represent individual DRG. Error bars reflect the SEM. *P* values represent one-way ANOVA with Tukey’s multiple-comparison test.(**D**) Representative photomicrographs of Masson’s trichrome–stained DRG obtained from mice treated with VS-4718 (*n* = 6) and selumetinib (*n* = 14), alone or in combination (*n* = 11) compared to age- and sex-matched vehicle controls (*n* = 6). Mask generated using Indica Lab’s HALO tissue area module is shown in the bottom panel and depicts collagen staining in purple. (**E**) Plot comparing the amount of collagen within the DRG, reflected as percentage of the total tissue area analyzed. Dots represent individual DRGs. Error bars reflect the SEM. *P* values represent one-way ANOVA with Tukey’s multiple-comparison test. *P ≤ 0.05, **P ≤ 0.01, ***P ≤ 0.001, ****P ≤ 0.0001, and n.s., not significant.

Given these results, we next evaluated the efficacy of VS-4718 plus selumetinib in vivo. Both single agent selumetinib and the combination of VS-4718 plus selumetinib significantly decreased DRG volume compared to VS-4718 and the vehicle control (Fig. 7A). Analysis of H&E-stained tissues using the Indica Labs HALO DenseNet AI module (Fig. 7, B and C) revealed a significant decrease in the percentage of abnormal tissue within the DRG of VS-4718- and combination-, but not selumetinib-, treated mice. Accordingly, analysis of trichrome staining demonstrated a significant decrease in collagen within DRGs from VS-4718- and combination-treated mice, but not those treated with selumetinib alone (Fig. 7, D and E). Collectively, these results suggest that combination VS-4718 plus selumetinib is more efficacious in reducing schwannoma tumor burden when compared to the use of either agent alone and support the evaluation of combination FAK and MEK inhibition for the treatment of NF2-SWN in future clinical trials.

## DISCUSSION

Recent work has suggested a role for FAK in schwannoma progression and development (*4*, *5*). In this study, we demonstrate that genetic ablation of *Fak* impairs schwannoma tumor formation and preserves hearing in a murine model of NF2-SWN. Through comprehensive molecular and cellular profiling, we provide previously unknown insight into the role of FAK in tumor cell intrinsic and microenvironmental processes. We demonstrate that the combined pharmacologic inhibition of FAK and MEK is synergistic and significantly reduces tumor burden in vivo. Our findings provide robust support for the evaluation of this combinatorial therapeutic strategy in future clinical trials for patients with NF2-associated schwannomas.

Prior studies have shown that Merlin, the protein product of *NF2*, negatively regulates FAK and that reexpression of exogenous Merlin attenuates FAK phosphorylation at tyrosine-397, a critical event for its full activation (*46*). Notably, work by Shapiro *et al.* (*47*) found that loss of *NF2* conferred increased sensitivity to FAK inhibition. Their work suggests that this synergy between *NF2* loss and FAK inhibition may be the result of weakened cell-cell adhesions occurring secondary to the loss of Merlin, which plays an important role in the establishment of stable adherens junctions (AJs) (*47*, *48*). The development of AJs has been shown to decrease the formation and reliance on focal adhesions (*49*, *50*). Consistent with this hypothesis, work by Flaiz *et al.* and Lallemand *et al.* (*51*, *52*) found that NF2-deficient SCs were characterized by impaired formation or stabilization of AJs. This loss of stable AJs following Merlin depletion drives an increased reliance on integrin-FAK signaling such that NF2-deficient tumors may become “addicted” to FAK for survival and thus more susceptible to FAK inhibition.

In addition to the disruptions in AJ development and stabilization, loss of *NF2* also promotes the proliferation and dedifferentiation of SCs to a nonmyelinating stage (*16*, *17*). These phenotypic changes are associated with myelination abnormalities and delayed regeneration of peripheral nerves following injury (*16*, *53*–*55*). Likewise, FAK is also known to play a role in SC differentiation, proliferation, and myelination (*19*, *56*). However, in contrast to *NF2* loss, *FAK*-deficient SCs undergo premature differentiation and exhibit reduced proliferation (*18*). This contrasting impact of *NF2* and *FAK* depletion in SCs may also explain the phenotypic differences observed in our *Nf2* and *DKO* mice. Unlike those obtained from *Nf2* mice, *DKO* DRGs were significantly smaller and exhibited significantly fewer onion-bulb formations, less collagen deposition, and fewer SCs, suggesting that in the setting of NF2 deficiency, genetic ablation of *Fak* promotes the normalization of SC proliferation and function.

Prior studies by our group and others suggest that schwannoma formation is driven by chronic inflammation and an unresolved SC-mediated repair response (*57*, *58*). Consistent with this hypothesis, evidence of defects in remyelination and a sustained inflammatory response has been observed within human schwannomas (*57*). More recent work further supports this hypothesis by suggesting that SCs with an injury-like phenotype drive the recruitment and infiltration of myeloid cells into the schwannoma TME (*33*, *34*). Macrophages play a major role in schwannoma pathogenesis, and their infiltration demonstrates a positive correlation with tumor growth and postoperative tumor progression (*59*–*62*). While the precise mechanisms by which macrophages contribute to the development and progression of SWN remain incompletely understood, compared to normal nerve, SWN are characterized by increased levels of inflammatory cytokines including TNFα and IL-1β (*63*, *64*). Through the analysis of publicly available scRNA-seq data of VS obtained from human patients, we found that VS-associated macrophages serve as the primary source of both TNFα and IL-1β. Upon binding to their receptors, TNFα and IL-1β can activate NF-κB, resulting in increased expression of NF-κB target genes including *NLRP3* and *IL6* (*29*). Notably, the increased expression of *IL-6* or *NLRP3* is associated with more severe hearing loss in human patients with VS (*65*–*69*). Here, we have shown that the genetic inhibition of *Fak* protects against the progressive hearing loss characteristic of the NF2 murine model and human patients suffering from VS. This improved hearing outcome was associated with decreased macrophage infiltration; suppression of TNFα, IL-1β, and NF-κB signaling; and a decrease in the expression of both IL-6 and *Nlrp3*. These findings are consistent with prior work demonstrating that FAK can directly regulate NF-κB activation and specifically does so during states of injury within the nervous system (*70*–*72*).

In addition to *IL6* and *NLRP3*, NF-κB also regulates the expression of *MET* and *HGF* (*30*). The HGF-MET axis plays an important role in the activation of SCs with a reparative phenotype during peripheral nerve injury, and the expression of both *MET* and *HGF* has been found to be significantly higher in VS compared to normal nerve (*30*, *35*, *73*–*76*). In addition, lower baseline levels of HGF are associated with an improved hearing outcome in response to bevacizuma,b and inhibition of MET using multi-RTK inhibitors suppresses the growth of human VS cells in vitro (*6*, *77*–*80*). Prior work suggests that a direct interaction between FAK and MET is thought to be required for specific HGF-mediated effects including cell motility and invasion (*77*). We observed suppression of the HGF-MET axis through multiple distinct mechanisms following *Fak* deletion in our *Nf2* mice. Compared to *Nf2* DRG, those obtained from *DKO* mice were characterized by a significant decrease in MET and a significant increase in the expression of *Spint1*, which encodes a protein known to prevent the activation and cleavage of pro-HGF. As FAK can directly regulate the activation of NF-κB, it is possible that this decrease in MET is a direct result of suppressed NF-κB–mediated transcriptional activity (*70*–*72*). Analysis of publicly available scRNA-seq data of VS obtained from human patients identified macrophages as the predominant source of HGF, suggesting that the decreased infiltration of macrophages observed in *DKO* DRG may be an additional mechanism by which *Fak* inhibition attenuates the HGF-MET signaling axis, thereby reducing tumor burden and preserving hearing.

While genetic ablation of *Fak* showed promising results, the pharmacological inhibition of FAK using VS-4718 as a single agent promoted the normalization of tissue architecture but was insufficient to reduce macroscopic tumor volume. The compensatory hyperactivation of the ERK signaling pathway observed in VS-4718–treated samples provided a potential explanation for this discrepancy and suggested that the combined use of FAK and MEK inhibitors may be required to achieve optimal therapeutic effect. These results are congruent with prior work demonstrating that FAK inhibition drives increased activation of the MAPK pathway (*43*, *71*). Subsequent in vivo experiments demonstrated that the dual targeting of FAK and MEK resulted in both a significant reduction in tumor volume and the preservation of DRG architecture. Our findings are consistent with recent studies demonstrating efficacy of dual FAK and MEK (with avutometinib/VS-6766) inhibition in metastatic melanoma models, including those resistant to standard of care B-Raf proto-oncogene, serine/threonine kinase inhibitor (BRAFi)/mitogen-activated protein kinase kinase inhibitor (MEKi) or immune checkpoint blockade (*44*, *81*). The clinical utility of this combinatorial strategy may offer several advantages over current therapeutic regimens for NF2-SWN. Brigatinib treatment led to volumetric reduction in 23% of tumors from patients with NF2-SWN, and while this is an important initial signature, most patients did not respond (*5*). In contrast, the targeted dual inhibition approach described here may provide enhanced therapeutic efficacy while maximizing tolerability. Our findings and those from others provide mechanistic insight into why single agent FAK or MEK inhibition alone may exhibit limited efficacy in NF2-SWN (*43*, *44*). The reciprocal activation between FAK and MAPK signaling suggests that monotherapy targeting of either pathway may be inherently limited by compensatory activation of the alternate pathway (*43*, *44*). By simultaneously targeting both pathways, this combinatorial approach addresses the underlying pathway cross-talk that may limit the efficacy of the respective monotherapies, potentially offering superior tumor control with improved tolerability compared to current treatment options. Accordingly, preclinical studies in multiple cancer types have driven active clinical trials targeting FAK and MEK in combination, particularly defactinib and avutometinib, which was recently granted FDA approval for use in ovarian cancer. Notably, in an early phase 1 clinical trial for patients with solid tumors, the use of combination defactinib plus avutometinib resulted in a 42% objective response rate (*82*). In our *Nf2* model, although the combination of VS-4718 and avutometinib was not well tolerated, inhibition of MEK using selumetinib showed promising results when combined with FAK inhibition, suggesting that the combination of selective FAK and MEK targeting drugs should be actively explored in the NF2-SWN setting.

Last, we observed differential effects between genetic and pharmacologic FAK inhibition, which highlight the complexity of FAK signaling in schwannoma development and progression. These differences may be attributed to several factors, including the timing of FAK inhibition (developmental versus post–tumor formation), the magnitude of inhibition achieved, and the potential role of kinase-independent functions of FAK. Several questions remain to be addressed in future studies. First, the precise mechanism by which FAK regulates macrophage recruitment and activation in the schwannoma microenvironment requires further investigation. Second, it is not entirely clear how FAK modulates the expression of MET. Several studies have shown a role for FAK in MET signaling, but none have demonstrated a direct regulatory role with respect to MET expression (*77*, *83*). Future studies further investigating the relative contributions the kinase-dependent and -independent functions of FAK will be important for gaining additional insights into its role in schwannoma genesis and treatment response. Nevertheless, the findings presented here provide ample support for the evaluation of combination FAK plus MEK inhibition in future clinical trials for patients with NF2-associated schwannomas.

## MATERIALS AND METHODS

### Study approval

All animal studies were conducted at the Indiana University School of Medicine in accordance with the Institutional Animal Care and Use Committee (IACUC) under protocol number 24020.

### Animal studies

#### Generation of mice and genotyping

*Nf2^flox/flox^;Postn-Cre^+^ (Nf2)* mice were generated in our laboratory as previously described (*12*). FAK^flox^ mice were purchased through the Jackson Laboratory (strain #: 031956, RRID:IMSR_JAX:031956) (*84*).Genotypes of each mouse were confirmed by polymerase chain reaction using genomic DNA obtained from ear snips and the primers described below.

1) *Nf2:* The *Nf2*^flox2^ alleles were detected as previously described by Giovannini *et al.* (*85*) and using primers P1 (5′-CTTCCCAGACAAGCAGGGTTC-3′) and P2 (5’-GAAGGCAGCTTCCTTAAGTC-3′), with bands detected at 442 base pairs (bp; *Nf2*^flox2^) and 305 bp (*Nf2*^+^).

2) Postn-Cre: The *Postn-Cre* transgene was detected using the following primers: P3 (5′-CATTTGGGCCAGCTAAACAT-3′) and P4 (5’-CCCGGCAAAACAGGTAGTTA-3′) with a band detected at 450 bp (Cre^+^) (*86*).

3) *Fak*: The *Fak*^flox^ alleles were detected using the primers P5 (5′-GAACTTGACAGGGCTGGTCT-3′) and P6 (5′-CTCCAGTCGTTATGGGAA ATCT-3′), yielding bands at 350 bp (mutant and flox/flox), 237 and 350 bp (heterozygote and flox/plus), and 237 bp (WT) (*84*).

#### Hearing assessment

Auditory brainstem responses (ABRs) were assessed at 4 and 10 months of age as we have previously described (*12*, *87*). Briefly, ABRs were measured in left and right ears of anesthetized mice [ketamine (100 mg/kg) and xylazine (10 mg/kg) by intraperitoneal injection] using subdermal needle electrodes. The Tucker-Davis Technologies (TDT) BioSigRZ system, with an RZ6 digital/analog converter (TDT), was used to produce and record responses to click stimuli [decibel sound pressure level (dB SPL)]. Click responses (512 repetitions, 30 to 90 dB SPL in 10-dB steps, and presented at 21/s with a closed-field system) were amplified, filtered (3 Hz to 3 kHz), averaged, and stored for offline analysis in MATLAB (MathWorks, Natick, MA, USA). ABR waveforms were high-pass filtered using a cutoff of 200 Hz to remove slow oscillations and emphasize characteristic ABR peaks. ABR threshold was defined as the lowest measured SPL at which a reproducible peak or trough was identified. Left and right ears were assessed independently and averaged to obtain an average ABR threshold (in decibels) for each mouse. Mice with an average ABR threshold value above 55 dB SPL at 4 months of age were considered hearing impaired at baseline and were excluded from further analysis (*WT*, *n* = 3; *Fak KO*, *n* = 0; *Nf2*, *n* = 1; *Nf2Fak^+/−^*, *n* = 1; *DKO*, *n* = 0) (*88*). Following the second ABR assessment at 10 months of age, mice were humanely euthanized, and tissues were harvested for downstream analyses. For mice in which no response was appreciated, a threshold of 90 dB SPL was recorded.

#### In vivo therapeutic studies

VS-4718 (PND-1186 and HY-13917) and selumetinib (AZD6244 and HY-50706) were purchased from MedChemExpress. Beginning at 4 months of age, *Nf2^flox/flox^;Postn-Cre^+^* (*Nf2*) mice were treated for 12 weeks with the maximum-tolerated doses of VS-4718 [25 mg/kg; oral gavage, bis in die; twice daily (BID)] and selumetinib (10 mg/kg; oral gavage, BID) alone or in combination. Two cohorts of age- and sex-matched *Nf2* mice administered the vehicle by oral gavage, twice daily, and served as controls. Animals were monitored daily for signs of distress such as hunched posture, labored breathing, scruffy coat, diarrhea, or lameness. All study mice began treatment at 4 months of age and were euthanized at the end of 12 weeks of treatment for histopathological assessment. Early end point criteria include a palpable mass of more than of 1500 mm^3^, greater than 20% reduction in body weight, any of the previously mentioned signs of distress that could not be remedied by the veterinary staff, or at the recommendation of the veterinarian. The total number of animals enrolled in each treatment group is as follows: vehicle: *n* = 26, VS-4718: *n* = 14, selumetinib: *n* = 14, and combination (VS-4718 plus selumetinib): *n* = 15. The number of animals that met the criteria for euthanasia and were humanely euthanized according to the IACUC protocol or that were found deceased before meeting criteria for euthanasia were as follows: vehicle: *n* = 2, VS-4718: *n* = 1, selumetinib: *n* = 2, and combination (VS-4718 plus selumetinib): *n* = 1. These mice were excluded from further analysis.

#### Tissue preparation and DRG volume quantification

Immediately following humane euthanasia, mice were fixed in 10% neutral buffered formalin. The bodies were decalcified in a 50:50 solution of 10% formic acid and 10% neutral-buffered formalin solution. DRGs were dissected microscopically, and the maximal dimensions (length and width) of four anatomically matched DRGs were determined using calipers. Volume was then approximated using the formula for the volume of a spheroid = 0.52 × (width)^2^ × length. For in vivo therapeutics studies, mice were euthanized after 12 weeks of treatment at approximately 7 months of age. For genetic intercross studies, two cohorts of mice were euthanized at 10 or 16 months of age.

### Histopathology

Tissues were microdissected from euthanized animals, fixed, and decalcified as described above. Specimens were then dehydrated with graded alcohols, cleared with xylenes, infiltrated with molten paraffin, and embedded in paraffin blocks. Four-micrometer-thick sections were cut on a Leica rotary microtome and stained with H&E or Masson’s trichrome. Tumors were assessed according to the established histological diagnostic criteria for schwannomas in human patients and murine genetically engineered murine models (GEMMs) as we have published previously (*12*, *30*, *87*). Abnormal tissue and nuclear quantifications were performed using H&E-stained slides and a custom or pretrained HALO AI DenseNet module, respectively (Indica Labs, version 4.0, RRID:SCR_018350). Quantification of trichrome staining was performed using the HALO Area Quantification module (Indica Labs, version 4.0). Area quantification analysis settings were optimized for the trichrome stain, and the intensity of staining in each annotated DRG was scored by the algorithm as negative (0; blue), weakly positive (1+; yellow), moderately positive (2+; orange), or strongly positive (3+; red). Data were exported to GraphPad Prism for statistical analysis as described below.

### Immunohistochemical staining

Five-micrometer-thick tissue sections were deparaffinized, hydrated, and transferred to 0.1 M EDTA (pH 9.0) for antigen retrieval in a pressure cooker. Samples were treated with 3% hydrogen peroxide for 10 min and then incubated with primary antibodies for 30 min at room temperature [F4/80: #70076, Cell Signaling Technology (1:100); IL-6: #bs0782R, Bioss (1:100); MET: #ab216574 or #ab51067, Abcam (1:100); SOX10: #ab180862, Abcam (1:100); NLRP3: #CAB12694, Assay Genie (1:1000 dilution); CD68, Cell Signaling Technology (1:100)]. Incubation with secondary antibody was performed for 30 min at room temperature [goat anti-rabbit, ab205718, Abcam (1:1000)], followed by 3,3′-Diaminobenzidine (DAB) (#8059, Cell Signaling Technology), which was applied for 10 min and terminated by rinsing in distilled water. Counterstaining was performed with Epredia Signature Series Hematoxylin 7211 (#22-050-111, Thermo Fisher Scientific), and the sections were dehydrated, cleared, and cover slipped. Slide images were acquired on an Aperio ScanScope CS at ×20 magnification. Quantitative immunohistochemical analyses for F4/80, IL-6, and SOX10 were conducted using the Cytonuclear IHC module of HALO Image Analysis software (Indica Labs, version 4.0). The intensity of nuclear staining was scored as negative (0; blue), weakly positive (1+; yellow), moderately positive (2+; orange), or strongly positive (3+; red). Quantitative immunohistochemical analyses for MET in the genetic model and IL-6 in the therapeutics model were conducted using the Area Quantification module of HALO Image Analysis software (Indica Labs, version 4.0). Quantitative immunohistochemical analyses for MET and CD68 in the therapeutic model were conducted using the multiplex IHC module of HALO Image Analysis software (Indica Labs, version 4.0). For all analyses, all intact DRGs on each slide were annotated to designate regions of interest, and analysis settings were optimized for each specific stain. On the basis of the degree of background staining, minimum optical density settings, and variations in staining intensity, either moderately plus strongly positive (2+ and 3+) or total positive (1+, 2+, and 3+) cells/tissues were used for statistical analysis as indicated in the figure legends. Data were exported to GraphPad Prism for statistical analysis as described below.

### IF staining

IF staining of F4/80 (#70076, Cell Signaling Technology; 1:100 dilution) and NLRP3 (#CAB12694, Assay Genie; 1:100 dilution) was performed using DRG from *Nf2* and *DKO* mice. Tissue sections were baked at 60°C for 1 hour, followed by deparaffinization in three changes of xylene and rehydration through a descending ethanol series. Slides underwent antigen retrieval using tris-EDTA (pH 9.0) in a pressure cooker at high pressure for 15 min. Sections remained in the antigen retrieval buffer and were allowed to cool for 1 hour before incubation in 3% hydrogen peroxide. Tissues were sequentially incubated with primary antibodies diluted in Tris-buffered saline with Tween-20 (TBST) for 1 hour at room temperature, followed by their respective fluorescent secondary antibodies ([F4/80: Goat anti-Rabbit IgG (H + L) Cross-Adsorbed Alexa Fluor 488, #A-11008, Invitrogen (1:2000); NLRP3: Goat anti-Rabbit IgG (H + L) Cross-Adsorbed Alexa Fluor 647, #A-21245, Invitrogen (1:2000)]. Slides were mounted with ProLong Diamond antifade mountant with 4′,6-diamidino-2-phenylindole (DAPI; #P36962, Invitrogen). Fluorescence signals were obtained using a DeltaVision Ultra microscope with a 60× lens (GE Healthcare, Chicago, IL, USA). Images were acquired with z-section of 0.2 μm each and deconvolved using SoftWoRx (GE Healthcare). Images were processed using Imaris (Bitplane, RRID:SCR_007370), with figures representing individual z-sections of deconvolved stacks.

### Western blot

Nerve tissue lysates were prepared as follows. Lysis buffer consisting of xTractor buffer (#635671, Takara Bio) plus cOmplete Mini Protease Inhibitor Cocktail (#11836153001, Roche) and PhosSTOP Phosphotase Inhibitor Cocktail (#4906837001, Roche) was added to each tissue sample, which were then subjected to sonication for 5 s at 70% AMPL three times and centrifugation for 20 min at 13,000 rpm. Protein concentrations were determined using the Pierce BCA Protein Assay Kit (#23227, Thermo Fisher Scientific). Isolated proteins were fractionated using NuPAGE 4–12% Bis-Tris Gels (#NP0322BOX, Invitrogen) and electrotransferred to polyvinylidene difluoride membranes. Immunoblots were carried out using antibodies specific to pERK (#9101, Cell Signaling Technology), total ERK (#9102, Cell Signaling Technology), phosphorylated FAK Y397 (pFAK 397, #700255, Invitrogen), or vinculin (#CP74, MilliporeSigma). Cell lysates were generated using MS03 cells treated with 1 μM VS-4718, selumetinib, or VS-4718 plus selumetinib in combination. At 24 hours, cell lysates were collected and prepared using lysis buffer consisting of 50 mM Hepes, 150 mM NaCl, 0.5% Triton X-100, 1 mM EDTA, and 1 mM EGTA (pH 7.5) supplemented with cOmplete Mini Protease Inhibitor Cocktail, 1% (v/v) Phosphatase Inhibitor Cocktails 2 and 3 (Sigma-Aldrich, #P5726 and #P0044), 10 mM NaF, and 2.5 mM NaVO_4_. Protein concentrations were determined using Pierce Coomassie (Bradford) Protein Assay Kit (PI23200, Thermo Fisher Scientific). Isolated proteins were fractionated using 4–20% Mini-PROTEAN TGX Precast Protein Gels (Bio-Rad, #4561094) and electrotransferred to nitrocellose membranes. Immunoblots were carried out using antibodies specific to pFAK 397 (#8556S, Cell Signaling Technology), total FAK (#3285S, Cell Signaling Technology), cleaved caspase 3 (#9664S, Cell Signaling Technology), and glyceraldehyde-3-phosphate dehydrogenase (GAPDH; #365062, Santa Cruz Biotechnology). Following incubation with primary antibody, blots were incubated with a horseradish peroxidase–conjugated secondary antibody (anti-rabbit: 7074 V or anti-mouse: 7076 V, Cell Signaling Technology). Signals were detected using enhanced chemiluminescence substrate (SuperSignal West Pico PLUS Chemiluminescent Substrate 34580 and/or SuperSignal West Femto Maximum Sensitivity Substrate 34095), and images were collected on a Bio-Rad ChemiDoc. Arbitrary densitometry units were calculated and quantified using the ImageJ software [National Institutes of Health (NIH), version 1.53], with signals normalized to the corresponding loading control and expressed relative to control conditions.

### Culture of cells

Human hTERT ipn02.3 2l SCs (“02.3”) were obtained from American Type Culture Collection (CRL-3392), and HEI-193 schwannoma (RRID:CVCL_7660) and murine (MS03 schwannoma) cell lines were generated as previously described (*89*, *90*). Cell lines were cultured in Dulbecco’s modified Eagle’s medium supplemented with 10% fetal bovine serum (Harvest MIDSCI), 1% glutamine (Gibco), 1% penicillin/streptomycin (Lonza), and prophylactic Plasmocin (5 μg/ml; Invivogen). The 02.3 cell line was transfected with equal amounts of a CRISPR-Cas9 KO plasmid pool targeting *NF2* and a homology-directed repair (HDR) plasmid pool purchased from Santa Cruz Biotechnology (sc-400504 and sc-400504-HDR, respectively). After selection with puromycin (2 ug/ml) and isolation of single-cell clones, multiple clones with complete KO of *NF2* were confirmed, and one used for further experiments (02.3 *NF2^−/−^* KO3). TrypLE Express Enzyme (Gibco) was used to dissociate cells for passaging upon reaching confluence. Cultures were tested for mycoplasma using the Lonza MycoAlert Mycoplasma Detection Kit and confirmed to be negative before experimentation.

#### Dose-response curves

For dose-response curves, cells were plated at a density of 5000 cells per well in 96-well plates and allowed to adhere overnight. Cells were then treated with increasing concentrations of defactinib (MedChemExpress, HY-12289) or FAK PROTAC Degrader 1 (MedChemExpress, HY-119932). Cell viability was assessed using the CellTiter-Glo Assay (Promega) 48 to 72 hours after treatment. End point luminescence was measured using a SynergyH4 plate reader with filters and settings as follows: 528/20 and hole filter sets, top read, 4-mm read height, gain: 135, and 0.5-s integration time.

#### Colony formation assays

For colony formation assays, 5000 cells were added to each well of a six-well plate and treated with the indicated drugs at the concentrations specified in the figure legends. DMSO served as the control. Cells were allowed to proliferate until controls were confluent (~5 to 7 days) and which time they were then stained with methylene blue diluted in 50% methanol and 50% H_2_O. For colorimetric measurement, each well was dissolved in 0.5 N of HCl, and the dissolved fluid was transferred to a 96-well plate to be read at 660 nm.

#### Synergy assays

MS03s were plated at a density of 2500 cells per well in 96-well plates and allowed to adhere overnight. Cells were then treated in a two-dimensional dose-response matrix of increasing concentrations of defactinib, selumetinib, or VS-6766 as indicated in the figure panels. At 48 hours posttreatment, cell viability was assessed using the CellTiter-Glo Assay (Promega). End point luminescence was measured using a SynergyH4 plate reader with filters and settings as follows: 528/20 and hole filter sets, top read, 4-mm read height, gain: 135, and 0.5-s integration time. Synergy graphs and Zero Interaction Potency score (ZIP), Loewe, and Bliss synergy scores were computed using the SynergyFinder package (version 3.2, RRID:SCR_019318) in R (*91*).

### Bulk RNA-seq of *Nf2* and *DKO* DRGs

Approximately eight DRGs per mouse were microdissected from each euthanized 10-month-old *Nf2* or *DKO* mouse and flash frozen using liquid nitrogen. Frozen DRGs from six mice per condition were sent to GENEWIZ from Azenta Life Sciences (South Plainfield, New Jersey, USA) for RNA extraction and bulk RNA-seq. Log_2_[counts per million (CPM) + 4] normalization of raw counts was performed using iDEP.96 (*92*). Plots for gene set enrichment (Figs. 2 and 3) and cell type mapping (reference dataset: LM22) were generated in Omics Playground software v2.7.18 run using a local Docker image as described by Akhmedov *et al.* (*93*). Plots for gene set enrichment in Fig. 4 were generated in R studio (RRID:SCR_000432). Statistical analysis was performed in R or using GraphPad Prism as detailed below.

### Drug-treated cell line bulk RNA-seq

Frozen cell pellets of murine *NF2*-deficient murine Schwannoma cells (MS03) (three replicates each) treated with defactinib (1 μM; MedChemExpress, HY-12289), VS-4718 (1 μM; MedChemExpress, HY-13917), GSK2256098 (1 μM; MedChemExpress, HY-100498), ifebemtinib (1 μM; IN-10018, MedChemExpress, HY-122844), PROTAC FAK Degrader 1 (1 μM; MedChemExpress, HY-119932), GSK215 (100 nM; MedChemExpress, HY-132296), or a FAK PROTAC kindly provided by B. Nabet (100 nM; BSJ-04-146) (*94*) were sent to GENEWIZ from Azenta Life Sciences (South Plainfield, New Jersey, USA) for RNA extraction and bulk RNA-seq. Log_2_(CPM + 4) normalization of raw counts was performed using iDEP.96 (*92*). Statistical analysis was performed in GraphPad Prism as detailed below.

### Human VS scRNA-seq

scRNA-seq data from three human schwannoma samples, generated by Baruah *et al.* (*95*), were downloaded from the Gene Expression Omnibus (GEO) under the accession number GSE250061. The filtered_bc_matrix.h5 files were read into R using the Read10X_h5 function in Seurat (version 4.0.2, RRID:SCR_016341). Seurat objects were created for each dataset with a minimum of three cells and 200 features per cell. Violin plots and scatter plots were generated to visualize the distribution of RNA features, counts, and mitochondrial content. Cells were filtered on the basis of the number of RNA features and mitochondrial content, retaining cells with more than 200 and fewer than 6000 RNA features and less than 20% mitochondrial content for AN014 and AN017 and fewer than 7000 RNA features for AN018. The datasets were then merged into a single Seurat object. The data were log normalized by a scaling factor of 10,000, and the top 2000 variable features were used for downstream principal components analysis, followed by Harmony integration to correct for batch effects. Thirty dimensions were used for identifying nearest neighbors, and clustering was performed at a resolution of 2. Uniform manifold approximation and projection (UMAP) was used for visualization. Marker genes for each cluster were identified using the FindAllMarkers function. Azimuth was used for cell type prediction, followed by supervised reannotation of cell types based on the expression of known marker genes (*96*).

GSEAs comparing MET+ and MET^–^ SCs were performed using the following packages: Seurat, msigdbr (version 7.4.1, RRID:SCR_022870), ggplot2 (version 3.3.5, RRID: SCR_014601), gridExtra (version 2.3), and enrichplot (version 1.14.0) (*97*, *98*). From the cell-type–annotated Seurat object generated above, data were preprocessed to combine SC subclusters into a single cluster (“Schwann”). SCs were then classified as either *MET*+ or *MET*^–^ based on *MET* gene expression greater than (*MET*^+^) or equal to (*MET*^–^) zero. DEG analysis was then performed to compare *MET*^+^ and *MET*^–^ populations using Seurat’s FindMarkers function. A min.pct = 0.1 and log fold change (FC) threshold = 0.25 were set to filter out genes with low expression levels and small FCs. GSEA was performed using the fgsea package (version 1.14.0, RRID:SCR_020938). The prepare_ranked_genelist function was used to create a ranked list of genes based on their average log_2_FC. The perform_gsea function was used to perform GSEA on the ranked list using the Hallmark gene sets or KEGG pathways from the Molecular Signatures Database with pathway size constraints of 15 to 500 genes. An enhanced GSEA visualization function (plot_pathway_enrichment_enhanced) was used to create mountain plots for the specific pathways. GSEA comparing NLRP3^+^ and NLRP3^–^ macrophages was executed similarly. Plot_gsea_results was used to generate the bar plot showing the top 20 pathways ranked by normalized enrichment score in Fig. 3K.

#### Population-based expression contribution analysis

For the population-based expression contribution analyses in Fig. 4 (C and D), cell type annotations were consolidated into broader categories by combining related subtypes to increase ease of interpretability: Nonmyelinating SCs (nmSC1 to nmSC5) were grouped as “nmSC,” myelinating SCs (mSC1 to mSC3) as “mSC,” dendritic cells (DC1 and DC2) as “DCs,” T cell subtypes (CD4 T, CD8 T, CD8 T Prolif, and other T) as “T cells,” and macrophage subtypes (Macrophage1 and Macrophage2) as “Macrophages.” Endothelial cells (Endo), B cells (B cells), vascular smooth muscle cells/pericytes (Peri/VS), fibroblasts (Fb), and natural killer (NK) cells remained as distinct categories. For each gene of interest (*MET* or *HGF*), the total expression within each cell type was calculated and normalized to the percentage of total expression across all cells using dplyr (RRID:SCR_022628). These population-level expression metrics were calculated to reflect the percentage contribution to the overall expression across all cell types and account for differences in cell type abundance. Visualizations were generated using ggplot2 and included bar plots showing the percentage of total gene expression contributed by each cell type.

#### CellChat analysis

CellChat (version 2.1.2, RRID:SCR_021946) was used to assess cell-cell communication within human VS (*99*). The normalized, log-transformed gene expression data matrix and assigned labels were extracted from the Seurat object generated above and used as input to construct a CellChat object. The human ligand-receptor CellChat database was subset to focus on secreted signaling pathways, and the data were preprocessed to identify overexpressed ligand-receptor interactions. The probability of communication between cell groups was computed using a truncated mean method (trim = 0.05) to build an intracellular communication network, which was extracted as a data frame for further analysis. Pathway-level communication probabilities were also computed and aggregated for downstream visualization of the HGF signaling pathway. A chord diagram was used to visualize communication dynamics between grouped cell types. A heatmap representation was used to depict the communication probability between various cell types. Last, centrality analysis was conducted to identify and visualize dominant signal senders, receivers, influencers, and mediators within the HGF pathway.

### Kinome profiling

MIB/MS was conducted to identify and quantitate the activity of the expressed kinome and their responses to drug treatment as previously described (*84*, *96*–*97*). Lysates prepared from NF2-deficient murine Schwannoma cell line (MS03) treated with 1 μM defactinib or VS-4718 for 24 hours were prepared and loaded onto the column containing multiple inhibitor-conjugated beads (*96*, *97*). In a separate experiment, the parental immortalized human SC line (02.3) and *NF2* knockout clones (described above) were harvested, lysed, and used for inhibitor bead enrichment. Kinase-bound inhibitor beads were stringently washed, followed by protein purification and trypsin digestion. Liquid chromatography, mass spectrometry, and analysis were performed essentially as previously described (*96*, *97*). Peptide suspension was separated using an EASY nLC-1200 System with an Easy-Spray C-18 column (Thermo Fisher Scientific). Raw files were processed for label-free quantification (LFQ) using MaxQuant LFQ and default parameters with the following modifications—razor plus unique peptides were used, matching between runs (3-min match time window), fixed modifications [carbidomethy (C)] and variable modifications [oxidation (M) and acetyl (protein N-terminal)], and searching the mouse (MS03) or human (02.3) UniProt databases (*98*). Kinase LFQ intensities were used if two or more unique + razor peptides were detected, and missing values were imputed in Perseus if observed in all replicates of at least one condition after log_2_ transformation for comparison. Data for each treated sample were plotted as a mean of FC (log_2_) relative to DMSO- or vehicle-treated control. When at least three replicates were available, unpaired Student’s *t* tests were performed in R. A *P* value of less than or equal to 0.05 was used as a cutoff for significance.

### Sciatic Nerve Atlas

#### Bulk RNA-seq of Nlrp3 expression across SC development

Bar plot of *Nlrp3* expression at respective stages of embryonic and postnatal development was obtained using bulk transcriptome analysis of fluorescence-activated cell sorting–isolated, P0Cre eYFP reporter–labeled SCs from the SNAT web portal (www.snat.ethz.ch) developed by Gerber *et al.* (*28*).

#### scRNA-seq data from the iSNAT

UMAPs depicting the expression of *Nlrp3*, *Hgf*, and *Met* at baseline and 1 or 3 days postinjury were obtained from the iSNAT web portal (https://cdb-rshiny.med.umich.edu/Giger_iSNAT/) developed by Zhao *et al.* (*36*). HGF signaling network data from 1, 3, and 7 days postinjury were also obtained from the iSNAT web portal using the CellChat module (*36*, *99*).

### Statistical analysis

Statistical analyses were performed in R studio (RRID:SCR_000432) or using GraphPad Prism 9.5.1 software (GraphPad, La Jolla, CA, RRID:SCR_002798). Specific analyses used to identify statistically significant differences between groups are detailed in the corresponding figure legends. *P* ≤ 0.05 were considered statistically significant for all analyses. For in vivo murine studies, a 5% type I error rate was assumed, and a power analysis was conducted to determine the appropriate sample size. Approximately *n* = 15 animals per group was determined necessary for the detection of a 40% difference in tumor size with greater than 85% power. Specific details related to individual analyses are described in the respective figure legends.
