# Supplementary material for: Inhibition of focal adhesion kinase impairs tumor formation and preserves hearing in a murine model of NF2-related schwannomatosis
Source: Sci Adv. 2026 Jan 30;12(5):eady8382. doi: 10.1126/sciadv.ady8382 (PMC12857737; doi:10.1126/sciadv.ady8382)
Supplement: Supplementary file 1 — Figs. S1 to S10 [file sciadv.ady8382_sm.pdf]

Science Advances
AAAS

Supplementary Materials for

Inhibition of focal adhesion kinase impairs tumor formation and preserves hearing in a murine model of NF2-related schwannomatosis

Dana K. Mitchell *et al.*

Corresponding author: Dana K. Mitchell, dm29@iu.edu; D. Wade Clapp, dclapp@iu.edu

Sci. Adv. **12**, eady8382 (2026)
DOI: 10.1126/sciadv.ady8382

This PDF file includes:

Figs. S1 to S10

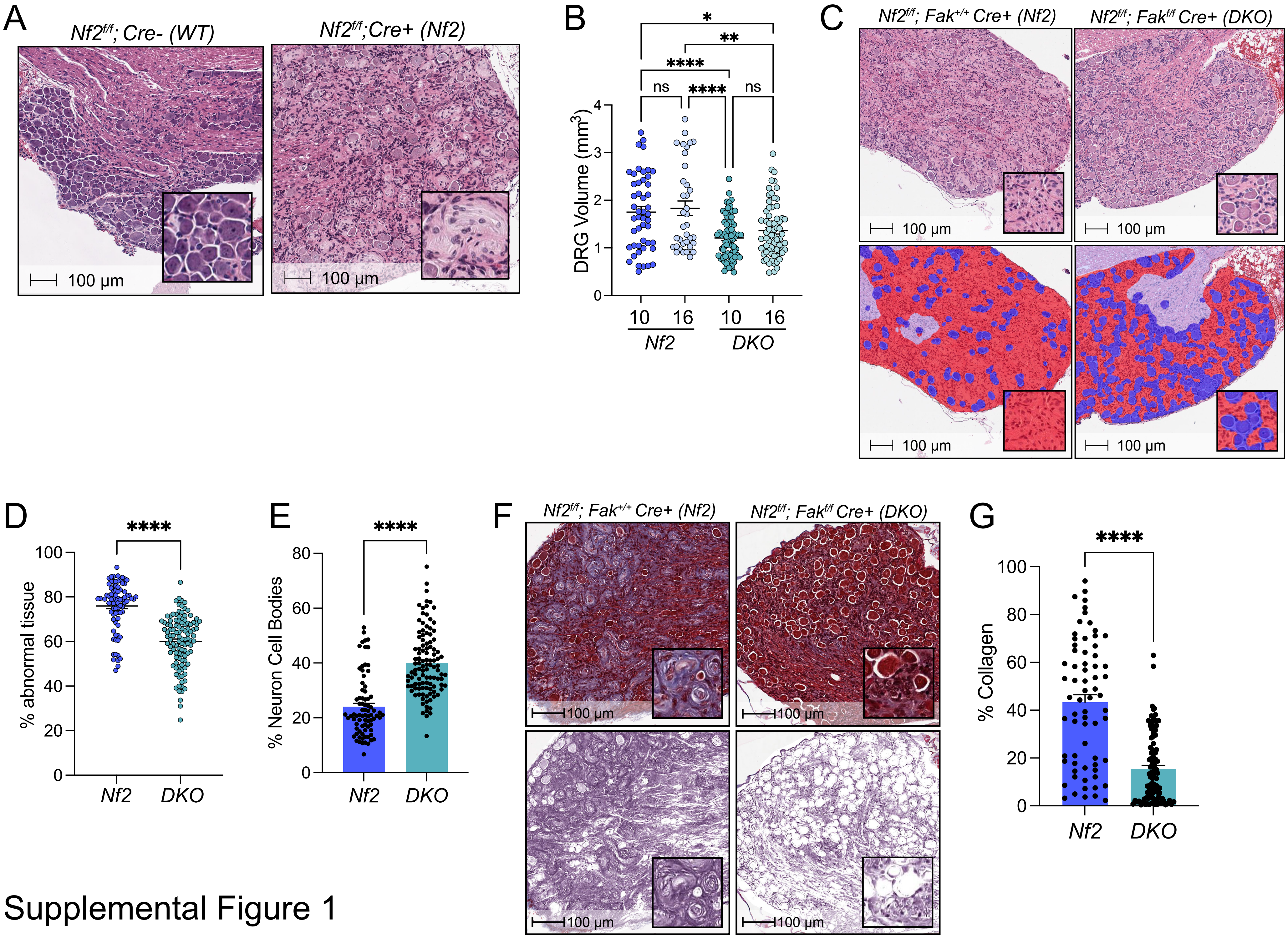

Supplemental Figure 1

Supplemental Figure 1. (A) Representative photomicrographs of H&E-stained slides comparing the tissue architecture of DRG obtained from WT (Left) and *Nf2* (Right) mice. Magnification denoted by 100 µm scale bars. **(B)** Scatter plot comparing dorsal root ganglion (DRG) volumes (mm^3) from 10- and 16-month-old *Nf2* (10m n=12, 16m n=9) and *DKO* (10m n=18, 16m n=17) mice. Dots represent individual DRG (n=4 per mouse). Error bars reflect the standard error of the mean (SEM). P-values represent one-way ANOVA with Tukey's multiple comparisons test. **(C)** Representative photomicrographs of H&E-stained DRG obtained from 16-month-old *Nf2* and *DKO* mice. Magnification denoted by 100 µm scale bars with inset high-power magnification as shown. Mask generated using Indica Lab's HALO AI DenseNet module is shown in the bottom panel and depicts abnormal tissue (red), neuron cell bodies (blue) and nerve (lavender). **(D,E)** Plots comparing the percentage of abnormal tissue **(D)** and neuron cell bodies **(E)** in 16-month-old *Nf2* and *DKO* DRGS**.** Dots represent individual DRGs. Error bars reflect the SEM. P-values represent unpaired, two-tailed t-tests between groups. **(F)** Representative photomicrographs of Masson's Trichrome-stained DRG obtained from 16-month-old *Nf2* and *DKO* mice. Magnification denoted by 100 µm scale bars with inset high-power magnification as shown. Mask generated using Indica Lab's HALO tissue area module is shown in the bottom panel and depicts collagen staining in purple. **(G)** Plot comparing the amount of collagen within the 16-month-old *Nf2* and *DKO* DRGs, reflected as percentage of the total tissue area analyzed. Dots represent individual DRGs. Error bars reflect the SEM. P-values represent unpaired, two-tailed t-tests between groups. ****=p≤0.0001, ***=p≤0.001, **=p≤0.01, *=p≤0.05, ns=not significant.

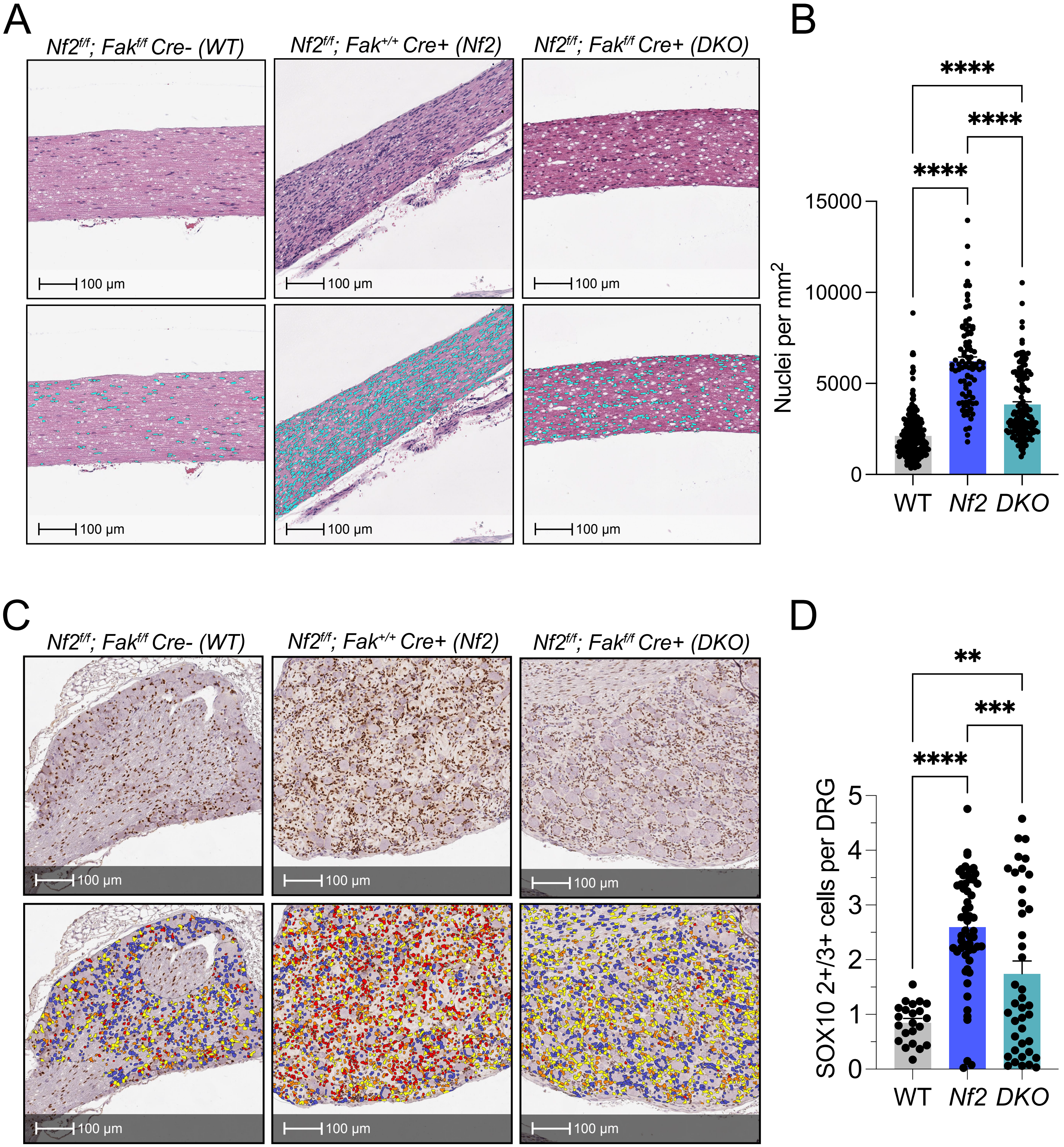

Supplemental Figure 2

Supplemental Figure 2. (A) Representative photomicrographs of H&E-stained nerves obtained from *WT*, *Nf2* and *DKO* mice. Magnification denoted by 100 µm scale bars. Mask generated using Indica Lab's HALO AI pre-trained nuclear (teal) detection module is shown in the bottom panel. **(B)** Plot comparing the number of nuclei per mm^2 in nerves from *WT*, *Nf2* and *DKO* mice**.** Dots represent individual regions of interest (ROIs). Error bars reflect the SEM. P-values represent one-way ANOVA with Tukey's multiple comparisons test. **(C)** Representative photomicrographs of nerves obtained from *WT*, *Nf2* and *DKO* mice and stained for SOX10. Magnification denoted by 100 µm scale bars. The HALO cytonuclear mask used to quantify the percentage of total cells exhibiting strong and moderate staining for SOX10 is shown in the bottom panel. Blue cells indicate negative staining for SOX10, yellow, weak positive (1+), orange, moderate positive (2+) and red, strong positive (3+) for SOX10 staining. **(D)** Plot depicting the percentage (%) of cells exhibiting 2+/3+ positive staining for SOX10 in DRGs from *WT*, *Nf2* and *DKO* mice**.** Dots represent individual regions of interest (ROIs). Error bars reflect the SEM. P-values represent one-way ANOVA with Tukey's multiple comparisons test. ****=p≤0.0001, ***=p≤0.001, **=p≤0.01, *=p≤0.05, ns=not significant.

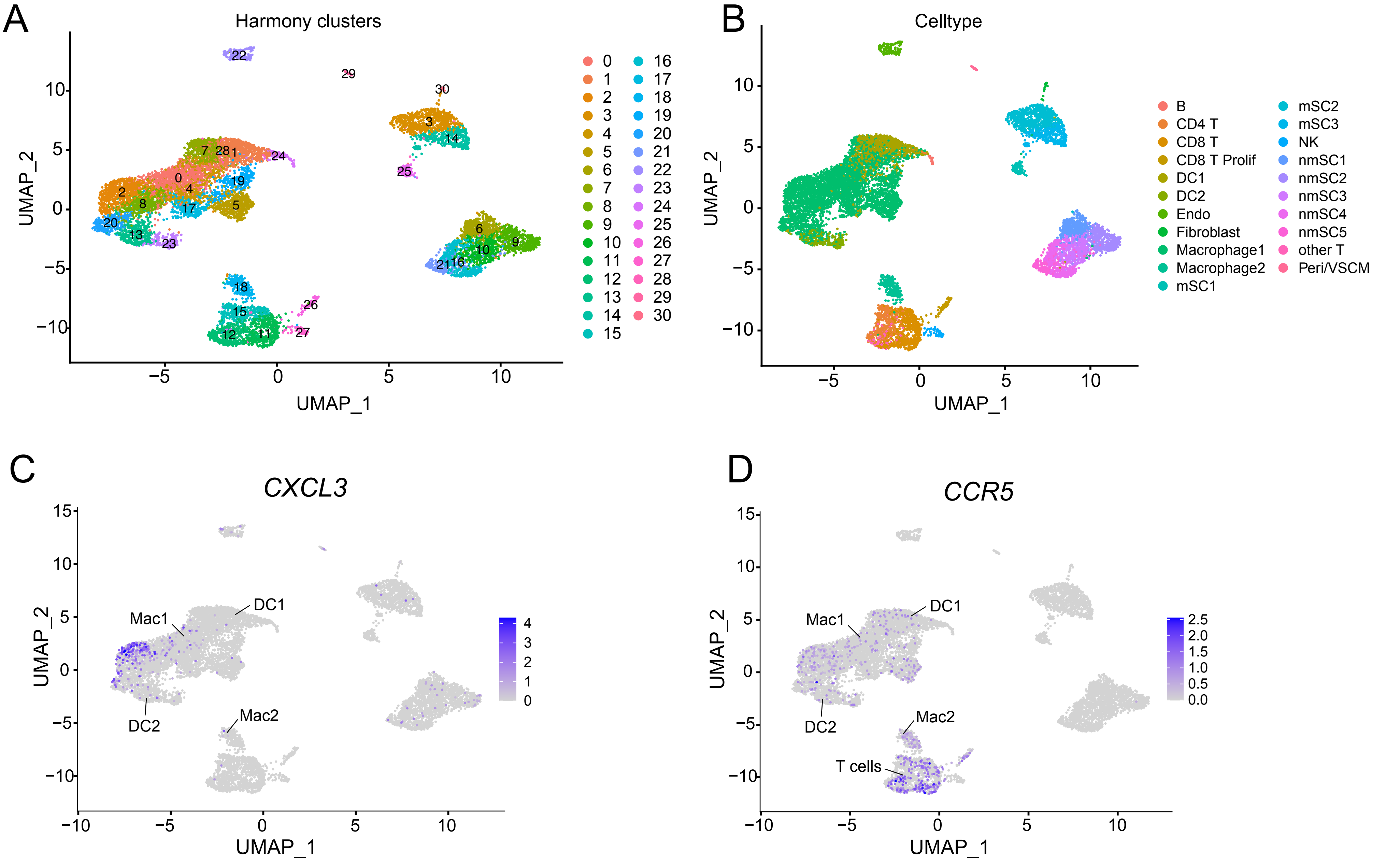

Supplemental Figure 3

Supplemental Figure 3. (A,B) UMAP plots of integrated single cell RNA sequencing data from human vestibular schwannoma samples annotated by cluster ID **(A)** and cell type **(B)**. **(C,D)** UMAP plots depicting the expression of *CXCL3* **(C)** and *CCR5* **(D)** across distinct cell populations in human vestibular schwannoma. Color overlays indicate transcript levels per cell. Gradient of grey to indigo indicates relative abundance of transcripts per cell, with indigo indicating higher expression and grey signifying a lack of detection. Mac1, Mac2 (macrophages), DC1, DC2 (dendritic cells), NK (natural killer cells), CD4 T ($CD4^+$ T cells), CD8 T ($CD8^+$ T cells), CD8 T Prolif (proliferating $CD8^+$ T cells), other T (other T cells), Endo (endothelial cells), mSC1-3 (myelinating Schwann cells), nmSC1-5 (non-myelinating Schwann cells), Peri/VSC (vascular smooth muscle cells/pericytes).

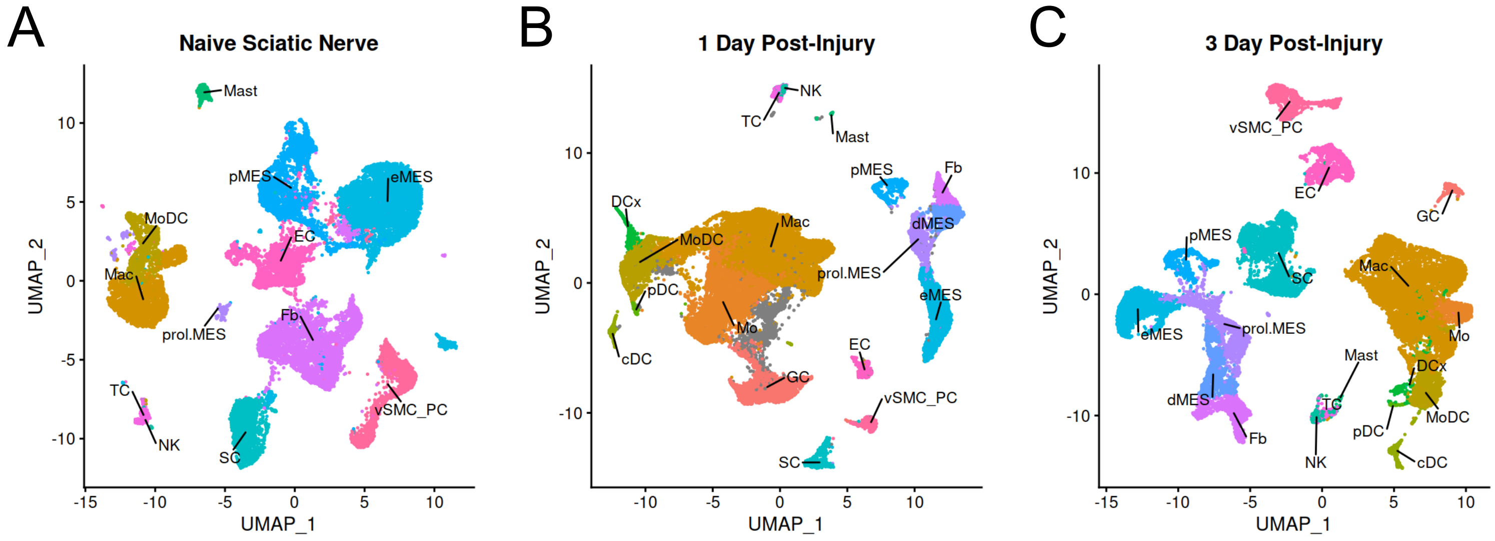

Supplemental Figure 4

Supplemental Figure 4. (A-C) UMAP plots obtained from the Injured Sciatic Nerve Atlas (iSNAT) of single cell RNA sequencing data from the naïve **(A),** post-injury day 1 **(B)** and post-injury day 3 **(C)** murine sciatic nerve. GC (granulocytes), Mo (monocytes), Mac (macrophages), MoDC (monocyte-derived dendritic cells), cDC (conventional dendritic cells), pDC (plasmocytoid dendritic cells), DCx (dendritic cells destined for homing), Mast (Mast cells), NK (natural killer cells), TC (T cells), eMES (endoneurial mesenchymal cells), pMES (perineurial mesenchymal cells), dMES (differentiating mesenchymal cells), prol.MES (proliferating mesenchymal cells), Fb (fibroblasts), SC (Schwann cells), EC (endothelial cells), vSMC_PC (vascular smooth muscle cells/pericytes).

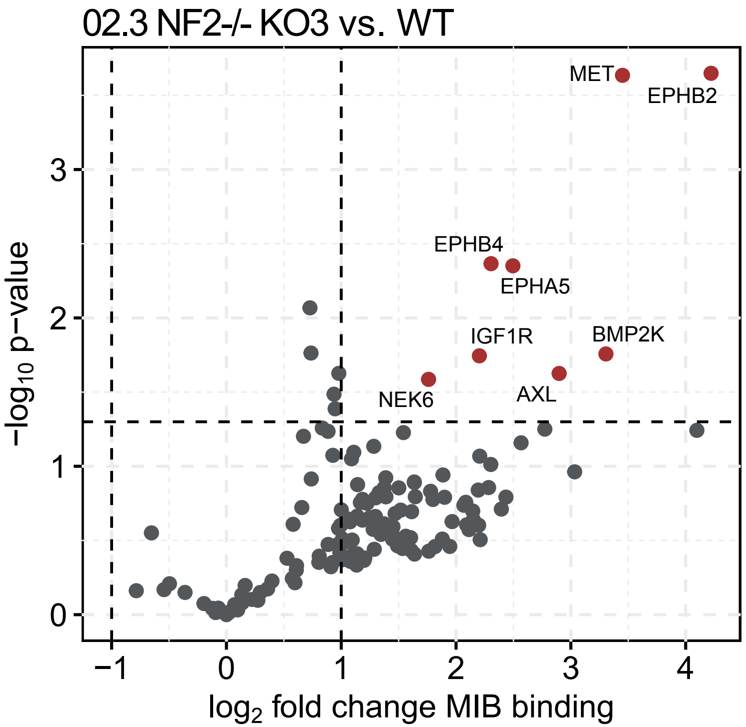

Supplemental Figure 5

Supplemental Figure 5. Volcano plot showing changes in multiplexed inhibitor beads (MIBs) binding of kinases in human NF2-deficient SCs compared to WT control. The x-axis represents log2 fold change in binding, and the y-axis shows the negative log10 of the p-value. Dotted lines indicate significance thresholds for fold change and p-value.

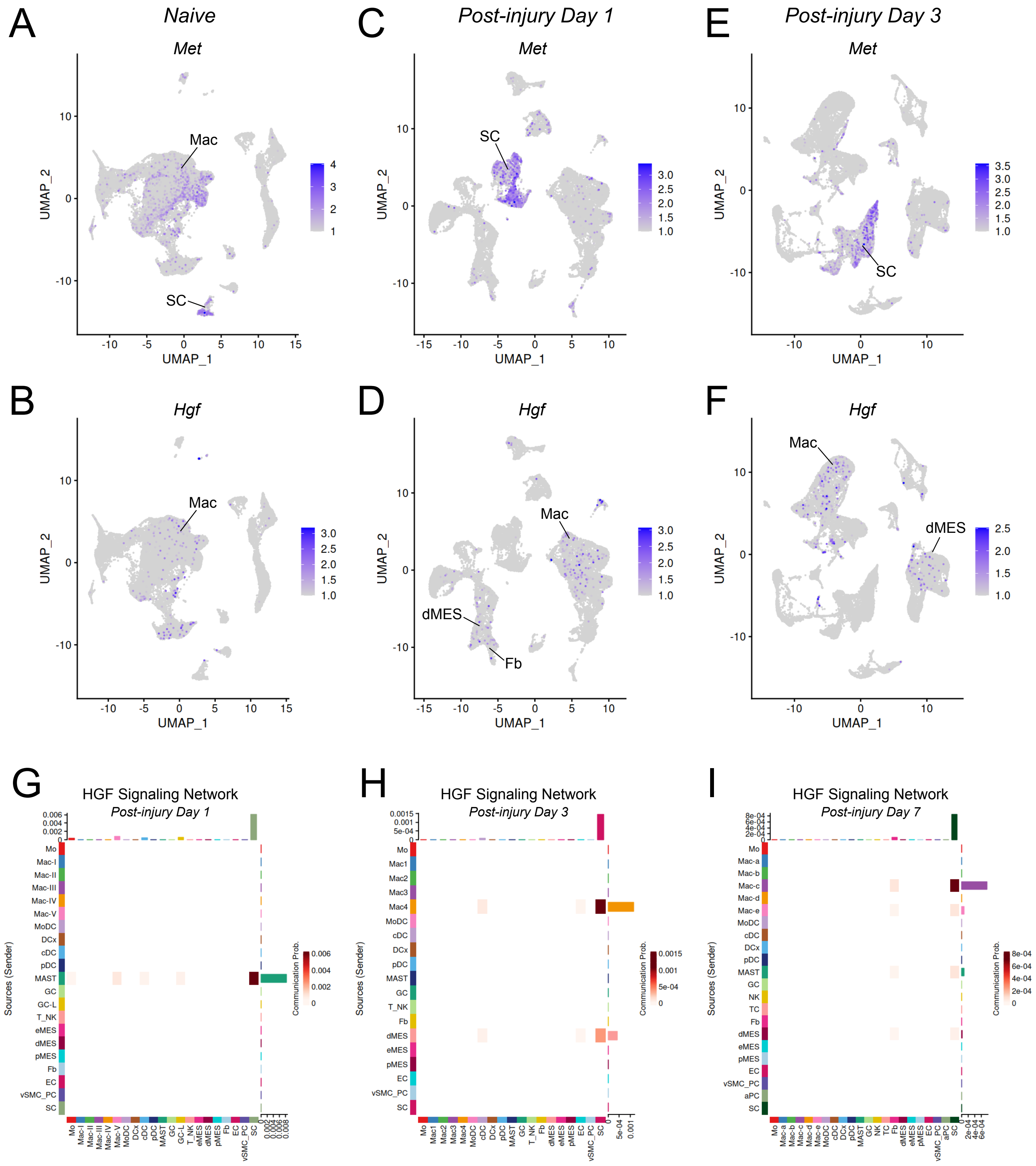

Supplemental Figure 6

Supplemental Figure 6. (A-F) UMAP visualizations obtained from the Injured Sciatic Nerve Atlas (iSNAT) depicting the expression of *Met and Hgf* across distinct cell populations in the murine sciatic nerve 1 **(C,D)** and 3 **(E,F)** days following injury**.** *Met and Hgf* expression in the naïve murine sciatic nerve serves as a reference **(A,B)**. Color overlays indicate transcript levels per cell. Gradient of grey to indigo indicates relative abundance of transcripts per cell, with indigo indicating higher expression and grey signifying a lack of detection. **(G-I)** Heatmaps obtained from the Injured Sciatic Nerve Atlas (iSNAT) depicting the communication probability scores between cell pairs within the HGF signaling network at 1 **(G)**, 3 **(H)** and 7 **(I)** days post-injury. Sender (source) cells are on the y-axis and receiver cells are on the x-axis. Gradient of white to maroon indicates the strength of predicted cell-cell communication with maroon indicating a high communication probability and white suggesting a lack of communication.

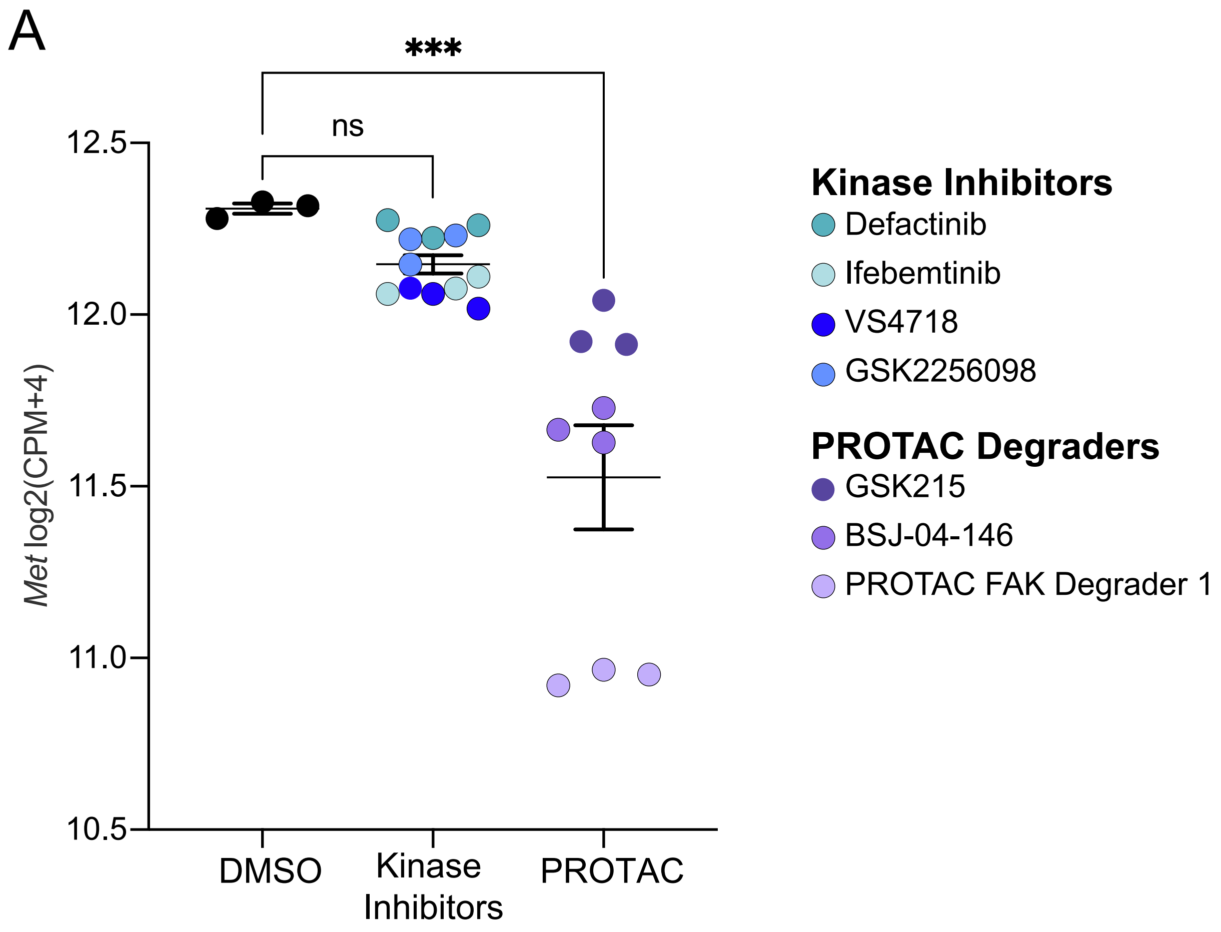

Supplemental Figure 7

Supplemental Figure 7. (A) Scatter plot comparing log_2 (CPM+4) normalized counts of *Met* expression in MS03 cells following treatment with 1 μM of FAK kinase inhibitors (defactinib, ifebemtinib, VS-4718 and GSK2256098) and FAK-targeting PROTACs (GSK215, BSJ-04-146, PROTAC FAK Degrader 1) compared to DMSO control. Dots represent independent replicates, with colors indicating the individual drugs as shown in the figure panel. Error bars reflect the standard error of the mean (SEM). P-values represent one-way ANOVA with Dunnett's multiple comparisons test. ****=p≤0.0001, ***=p≤0.001, **=p≤0.01, *=p≤0.05, ns=not significant.

Supplemental Figure 8

A

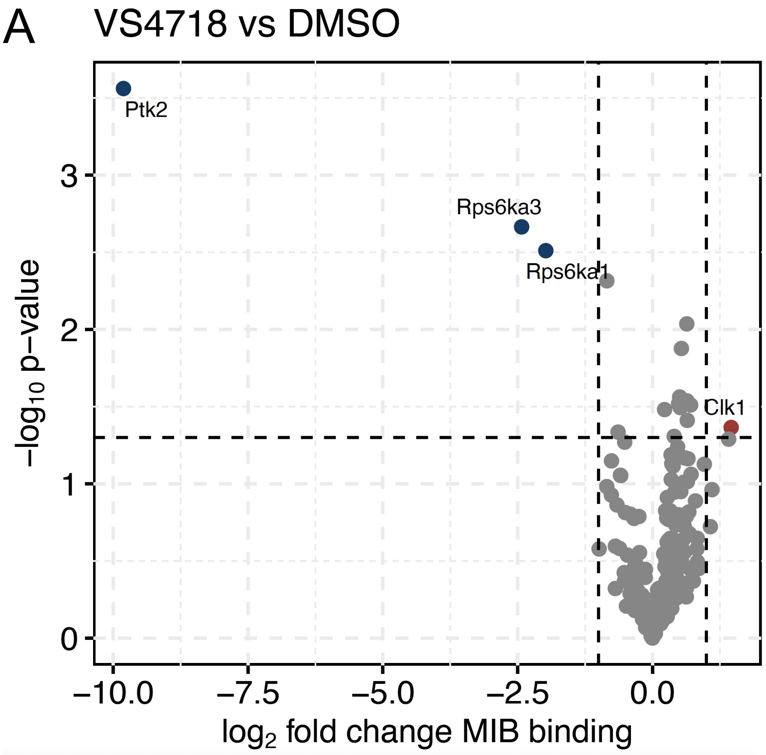

Supplemental Figure 8. (A) Volcano plot showing changes in multiplexed inhibitor beads (MIBs) binding of kinases following 24hr treatment with VS-4718 compared to DMSO control in MS03 cells. The x-axis represents log2 fold change in binding, and the y-axis shows the negative log10 of the p-value. Dotted lines indicate significance thresholds for fold change and p-value.

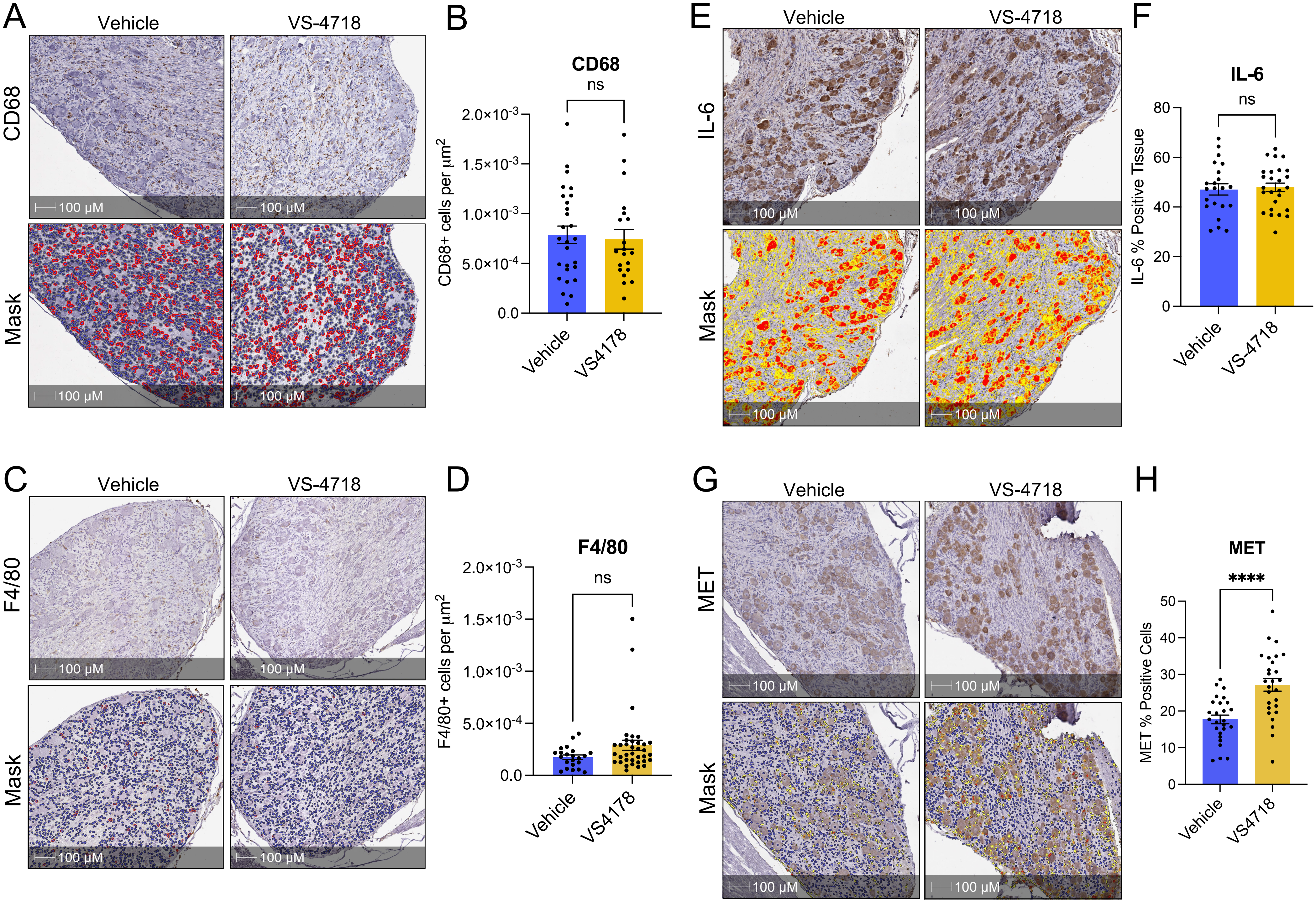

Supplemental Figure 9

Supplemental Figure 9. (A-D) Representative photomicrographs of vehicle and VS-4718 treated DRG immunohistochemically stained for macrophage markers CD68 **(A)** and F4/80 **(C)**. Magnification is denoted by 100 μm scale bars with inset high-power magnification as shown. The HALO multiplex IHC cytonuclear mask used to quantify the number of cells per μm^2 positively stained for CD68 or F4/80 is shown in the bottom panel. Yellow indicates weak positive (1+), orange, moderate positive (2+) and red, strong positive (3+). **(B,D)** Bar graphs depicting the number of CD68+ **(B)** and F4/80+ **(D)** cells per μm^2 in DRG obtained from vehicle and VS-4718 treated mice. Dots represent individual DRGs. Error bars reflect the standard error of the mean (SEM). P-value represents unpaired, two-tailed t-tests between groups. **(E)** Representative photomicrographs of vehicle and VS-4718 treated DRG immunohistochemically stained for IL-6. Magnification is denoted by 100 μm scale bars with inset high-power magnification as shown. The HALO area mask used to quantify the percentage of tissue area positively stained for IL-6 is shown in the bottom panel. Yellow indicates weak positive (1+), orange, moderate positive (2+) and red, strong positive (3+). **(F)** Bar graph depicting the percentage of IL-6 positive tissue in DRG obtained from vehicle and VS-4718 treated mice. Dots represent individual DRGs. Error bars reflect the standard error of the mean (SEM). P-value represents unpaired, two-tailed t-tests between groups. **(G)** Representative photomicrographs of vehicle and VS-4718 treated DRG immunohistochemically stained for MET. Magnification is denoted by 100 μm scale bars with inset high-power magnification as shown. The HALO multiplex IHC cytonuclear mask used to quantify the percentage of cells positively stained for IL-6 is shown in the bottom panel. Yellow indicates weak positive (1+), orange, moderate positive (2+) and red, strong positive (3+). **(H)** Bar graph depicting the percentage of MET positive cells in DRG obtained from vehicle and VS-4718 treated mice. Dots represent individual DRGs. Error bars reflect the standard error of the mean (SEM). P-value represents unpaired, two-tailed t-tests between groups. ****=p≤0.0001, ***=p≤0.001, **=p≤0.01, *=p≤0.05, ns=not significant.

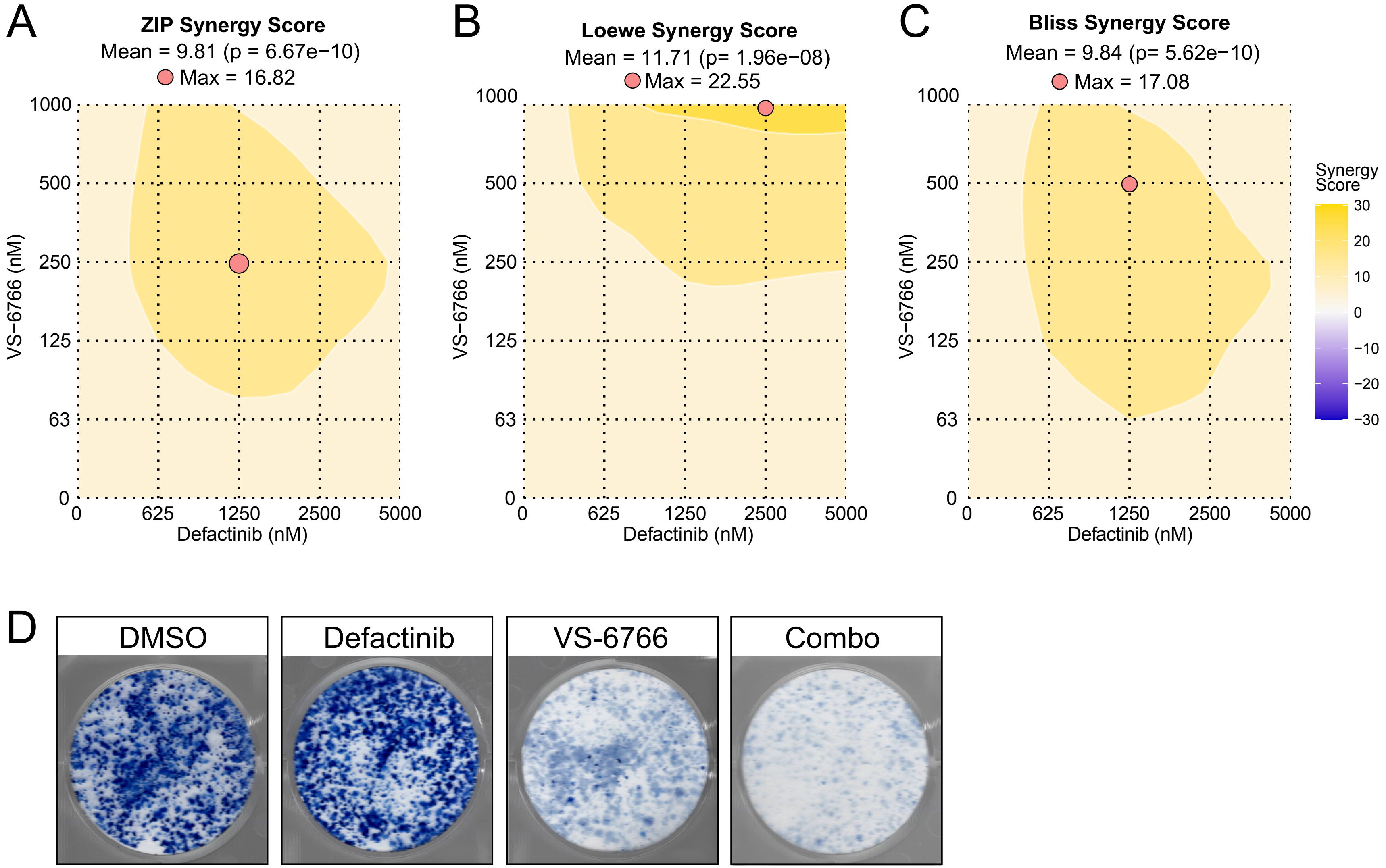

Supplemental Figure 10

Supplemental Figure 10. (A-C) Heatmaps depicting ZIP **(A),** Loewe **(B),** and Bliss **(C)** synergy scores for combination defactinib plus RAF/MEK inhibitor VS-6766 in MS03 cells. Viability (%) was evaluated at 48 hours by CellTiter-Glo® and normalized to the untreated control. Color scale indicates synergy scores from -30 (blue, antagonistic) to +30 (yellow, synergistic). Heatmaps, mean synergy scores and corresponding statistical significance (ZIP: 16.82, $p = 6.67e-10$; Loewe: 11.71, $p = 1.96e-08$; Bliss: 9.84, $p = 5.62e-10$) were computed using the SynergyFinder package in R. **(D)** Colony formation assay stained with methylene blue showing the effect of 6 days of treatment with combination defactinib (1 μM) plus RAF/MEK inhibitor VS-6766 (1 μM) compared to single agent defactinib (1 μM) or VS-6766 (1 μM) and DMSO control in the MS03 cell line.
